# Research progress on the mechanism of curcumin anti-oxidative stress based on signaling pathway

**DOI:** 10.3389/fphar.2025.1548073

**Published:** 2025-04-07

**Authors:** Jie Cui, Haonan Li, Tianyi Zhang, Fengli Lin, Meiyun Chen, Guimin Zhang, Zhong Feng

**Affiliations:** ^1^ School of Pharmacy, Shandong University of Traditional Chinese Medicine, Jinan, China; ^2^ Graduate School, Tianjin University of Traditional Chinese Medicine, Tianjin, China; ^3^ School of Pharmaceutical Sciences (Shenzhen), Sun Yat-sen University, Shenzhen, China; ^4^ Lunan Pharmaceutical Group Co., Ltd., Linyi, China

**Keywords:** curcumin, oxidative stress, Keap1-Nrf2/ARE, NF-κB, NOX, MAPK

## Abstract

Oxidative stress refers to an imbalance between oxidative capacity and antioxidant capacity, leading to oxidative damage to proteins, lipids, and DNA, which can result in cell senescence or death. It is closely associated with the occurrence and development of various diseases, including cardiovascular diseases, nephropathy, malignant tumors, neurodegenerative diseases, hypertension, diabetes, and inflammatory diseases. Curcumin is a natural polyphenol compound of β-diketone, which has a wide range of pharmacological activities such as anti-inflammatory, antibacterial, anti-oxidative stress, anti-tumor, anti-fibrosis, and hypolipidemic, demonstrating broad research and development value. It has a wide range of biological targets and can bind to various endogenous biomolecules. Additionally, it maintains the redox balance primarily by scavenging ROS, enhancing the activity of antioxidant enzymes, inhibiting lipid peroxidation, and chelating metal ions. This paper systematically describes the antioxidative stress mechanisms of curcumin from the perspective of signaling pathways, focusing on the Keap1-Nrf2/ARE, NF-κB, NOX, MAPK and other pathways. The study also discusses potential pathway targets and the complex crosstalk among these pathways, aiming to provide insights for further research on curcumin’s antioxidant mechanisms and its clinical applications.

## Graphical Abstract

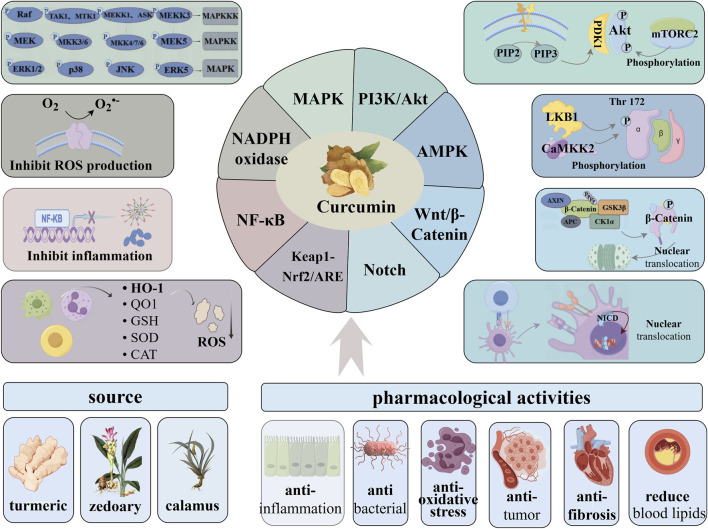

## 1 Introduction

Oxidative stress refers to an imbalance between oxidative capacity and antioxidant capacity, primarily due to the disruption of redox signaling. This condition is closely associated with either the excessive production of reactive oxygen species (ROS) or the diminished efficacy of the body’s antioxidant defense mechanisms (Ji and Yeo, 2021). ROS are derivatives of oxygen molecules produced during aerobic metabolism. Normal levels of ROS play an important role in intracellular signal transduction, immunity, metabolism, hypoxia response and transcriptional regulation, and participate in various cellular functions based on redox regulation (Rhee, 2006; Kishi et al., 2024; Forrester et al., 2018). Excessive ROS is pathological and prone to oxidative damage to proteins, lipids and DNA, resulting in cell senescence or death. Among them, protein is the main damage target, which may change its structure, function and turnover rate, resulting in loss or occasional increase in its activity (Hawkins and Davies, 2019). Furthermore, high levels of ROS can activate inflammation-related transcription factors such as NF-κB and AP-1, promoting the expression of inflammatory mediators and triggering inflammatory responses (Schieber and Chandel, 2014). During the inflammatory process, neutrophils and other immune cells activate a powerful oxidative burst, producing a large number of superoxide radicals. This further aggravates oxidative stress, forming a vicious cycle that exacerbates disease progression. (Winterbourn et al., 2016). There are many factors that cause cellular oxidative stress. Endogenous processes mainly focus on resistance to pathogens and enzymatic reactions, while exogenous factors include radiation, pollutants, drugs or chemicals (Hawkins and Davies, 2019), which are conducive to the occurrence and development of chronic diseases such as cardiovascular diseases, nephropathy, malignant tumors, neurodegenerative diseases, hypertension, diabetes and inflammatory diseases (Forrester et al., 2018; Sies et al., 2017).

**FIGURE 1:**
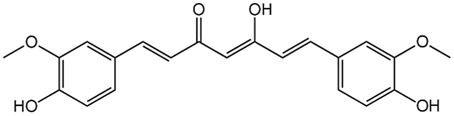
Chemical structure of curcumin.

At present, a series of natural plant active ingredients have been studied and developed into mature antioxidant drugs, such as ginsenosides (Zhou et al., 2022), astragaloside (Xing et al., 2021), notoginsenoside (Li X. et al., 2021) etc., all of which have shown good therapeutic effects. Curcumin, a natural polyphenol compound, is primarily extracted from the roots of turmeric (Curcuma longa). While also found in smaller amounts in other Curcuma species like zedoary and calamus, turmeric remains the primary source. It tastes slightly bitter and is insoluble in water. Its chemical structure is shown in Figure 1. It has a wide range of pharmacological activities such as anti-inflammation, antibacterial, anti-oxidative stress, anti-tumor, anti-fibrosis, lowering blood lipids, etc., showing broad research and development value (Patel et al., 2020). It is highly efficient and low toxic, and its β-dione and phenyl polyconjugated structures contribute to electron delocalization above the hydroxyl oxygen atom of phenol or provide H atoms to directly neutralize reactive oxygen species. It has a wide range of biological targets and can bind to a variety of endogenous biomolecules, including enzymes, receptors, signaling molecules, metals, transcription factors, and even some proteins located in cell membranes (Hatamipour et al., 2018). It maintains the redox balance of the system primarily by scavenging ROS, enhancing the activity of antioxidant enzymes, inhibiting lipid peroxidation, and chelating metal ions (Farzaei et al., 2018). Moreover, curcumin interacts with a variety of metabolic processes to exert its extensive biological effects. In glucose metabolism, it improves insulin sensitivity and inhibits gluconeogenesis, thereby reducing blood sugar levels (Ren et al., 2020). In lipid metabolism, it promotes the oxidation of fatty acids and reduces blood lipids (Silva-Gaona et al., 2022). In energy metabolism, it improves mitochondrial function and enhances ATP production (Chen et al., 2019). Finally, it regulates intestinal microbial composition, promotes the production of short-chain fatty acids, and improves intestinal health (Chang et al., 2023). The aim of this paper is to systematically elucidate the molecular mechanism of curcumin anti-oxidative stress from the perspective of signaling pathway, and discuss the potential pathway targets.

## 2 Keap1-Nrf2/ARE signaling pathway

**FIGURE 2:**
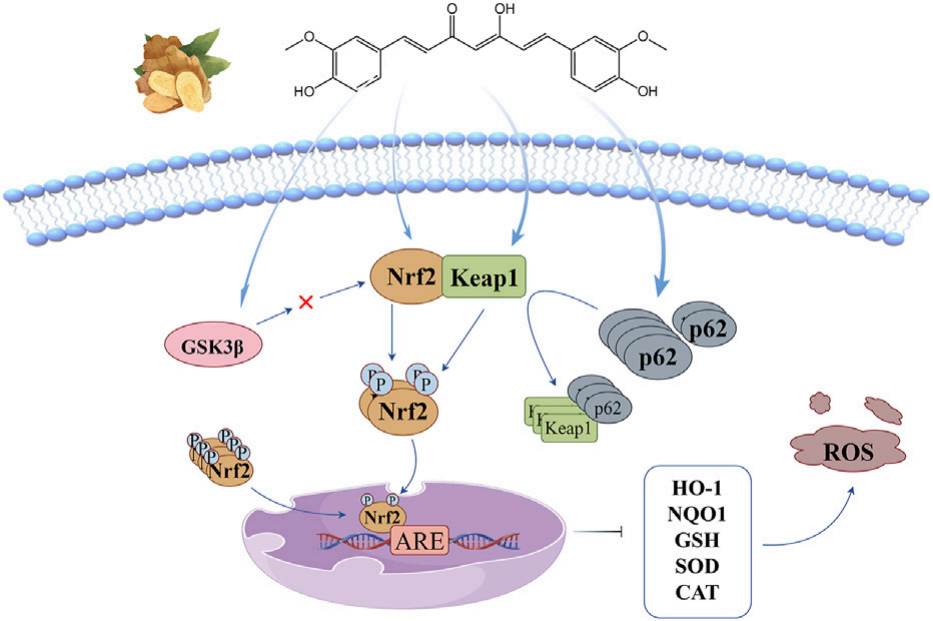
Keap1-Nrf2/ARE signaling pathway: curcumin promotes Nrf2 nuclear translocation by inducing threonine and serine phosphorylation of Nrf2, changing Keap1 conformation, promoting p62 accumulation, and inhibiting GSK3β activity.

Nuclear factor-E2-related factor-2 (Nrf2) is a major regulatory transcription factor of antioxidant genes, controlling the expression of a series of antioxidant enzymes, detoxification enzymes and other cellular defense and repair proteins. Its activation is beneficial to enhance the endogenous antioxidant system of tissues, inhibit the inflammatory signaling cascade and gene-induced apoptosis pathway (Hamdy et al., 2024). The anti-oxidant stress mechanism of curcumin based on this signaling pathway is illustrated in Figure 2. In homeostatic equilibrium state, Nrf2 binds to Kelch-like epichlorohydrin-associated protein 1(Keap1) in cytoplasm to form a stable complex, and relies on ubiquitination and proteasomal degradation to maintain its low expression level. Under the stimulation of oxidative stressors, the stability of Nrf2 in the cytoplasm is enhanced and transferred into the nucleus, where it binds to antioxidant response elements (ARE) in the promoter region of antioxidant oxidase gene Ⅱ to initiate the transcription of corresponding target genes (Niture et al., 2010), induce the production of antioxidant enzymes such as heme oxygenase-1 (HO-1), NADPH quinone reductase-1 (NQO1), glutathione (GSH), superoxide dismutase (SOD) and catalase (CAT), and maintain the system’s redox homeostasis (Yang et al., 2019).

Keap1-Nrf2/ARE signaling pathway is currently the most critical endogenous antioxidant stress pathway, and it is generally believed that curcumin promotes Nrf2 nuclear translocation through four pathways: (1) Curcumin directly induces the phosphorylation of threonine (Thr) and serine (Ser) of Nrf2, promotes the release of Nrf2 from the stable complex, and accelerates its nuclear translocation (Pulido-Moran et al., 2016); (2) α, β unsaturated carbonyl of curcumin covalently binds to Keap1 cysteine residue through Michael addition, causing conformation changes in Keap1, resulting in Nrf2 dissociation from the stable complex and transfer into the nucleus. However, Shin et al. (2020) believe that the increase of nuclear expression of Nrf2 does not depend on complex dissociation, but blocks the ubiquitin-mediated degradation pathway of Keap1 effectively targeting Nrf2 by changing the conformation of Keap1, thus promoting the newly synthesized Nrf2 to enter the nucleus. (3) p62 is an adapter protein in the ubiquitination system, and curcumin induces its accumulation and its interaction with Keap1 to promote the nuclear translocation of Nrf2 (Li et al., 2023); (4) There is a negative feedback regulation between glycogen synthase kinase 3β (GSK3β) and Nrf2, Curcumin can promote the dissociation of Nrf2 from the complex by inhibiting the activity of GSK3β, and then transfers into the nucleus (Li et al., 2020b).

The loss of neurons caused by oxidative stress is a key factor in the increased disability rate of cerebral hemorrhage (ICH). Duan et al. (2022) established a rat ICH model by intracavity injection of autologous blood, which was accompanied by severe intracranial oxidative stress and secondary nerve function injury. Curcumin treatment effectively activated Nrf2 signaling pathway, upregulated the expression of HO-1, NQO1, Gpx4 and other antioxidant proteins, significantly reduced the ROS and malonaldehyde (MDA) levels in the surrounding tissue of intracranial hematoma in rats, and effectively promoted the clearance of intracranial hematoma in rats. Moreover, the inhibitory effect of Nrf2 inhibitor ML385 on neural function recovery confirmed the key role of Nrf2/HO-1 pathway in curcumin’s prevention and treatment of ICH. Diabetic cardiomyopathy is one of the most common complications of diabetes mellitus, mainly manifested by cardiac microvascular disease and metabolic dysfunction. Wu et al. (2022) found that curcumin treatment significantly alleviated the decline in the viability of H9C2 cardiocytes induced by high glucose stimulation, promoted the nuclear accumulation of Nrf2 protein, enhanced the activities of antioxidant enzymes HO-1, SOD and Gpx, and inhibited the production of ROS. The inhibitory effect of shRNA-Nrf2 on the above functions further verified the key role of curcumin in activating Nrf2/HO-1 pathway in myocardial protection, and curcumin intervention significantly reduced the lipid accumulation in myocardium of streptozotocin (STZ) -induced rats with type 2 diabetes, inhibited myocardial remodeling and apoptosis, and improved lipid metabolism and myocardial function. Epithelial-mesenchymal transition (EMT) is one of the important mechanisms of myocardial fibrosis. Chen et al. (2022) showed that in a rat model of cardiac fibrosis induced by subcutaneous injection of isoproterenol, Curcumin treatment effectively activated the Nrf2-NO pathway, significantly enhanced the activity of nitric oxide synthase (eNOS), upregulated the expression of endothelial cell markers, but downregulated the expression of interstitial cell markers α-SMA and vimentin, significantly restored endothelial cell function, improved cardiomyocyte hyperplasia and inhibited the EMT process of cardiac fibrosis. In a mouse model of kidney injury induced by aflatoxin B1(AFB1) (Wang et al., 2022), oral curcumin can significantly increase the expression levels of Nrf2 and its downstream genes CAT, SOD1, NQO1 and glutamate-cysteine ligase (GCLC), inhibit the apoptosis mediated by Bax/Bcl-2-Cyt-c signaling cascade, and antagonize the renal toxicity caused by AFB1. Xu et al. (2024) found in a mouse model of lung injury induced by water-based arsenic poisoning that curcumin could not only accelerate the metabolism of arsenic and significantly improve lung tissue morphology, but also promote the expression and nuclear translocation of Nrf2 protein, increase the expression of its downstream related proteins such as NQO1, GCLC and HO-1, enhance the body’s endogenous antioxidant capacity and promote cell survival. Guo et al. (2021) found that curcumin intervention alleviated T-BHP-induced oxidative damage of human corneal endothelial cells (CECs), maintaining the original form of the cells and promoting their proliferation and differentiation. It is worth noting that high concentrations of curcumin (>50 μM) will induce the production of a large number of ROS and even cause DNA damage. Dysregulation of the Nrf2 pathway is a contributor to depression/neurodegenerative diseases. Long-term curcumin administration can activate Nrf2 nuclear translocation and its downstream pathway, effectively reduce MDA, 4-hydroxynonenal (4-HNE) and serum corticosterone levels, successfully reverse the increased expression of 8-hydroxy-2 ′-deoxyguanosine (8-OHDG) under chronic and unpredictable mild stress, reduce DNA oxidative damage, and significantly improved depression-like behavior in rats (Liao et al., 2020).

## 3 NF-κB signaling pathway

**FIGURE 3:**
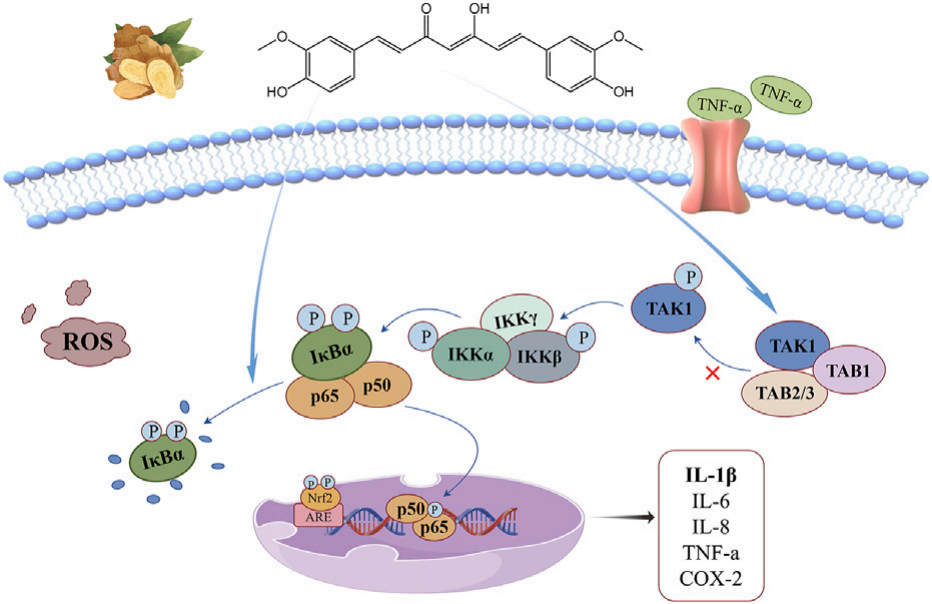
NF-κB signaling pathway: curcumin can reduce the interaction between TAK1 and TAB1, inhibit the degradation of IκBα in cytoplasm, and up-regulate antioxidant gene expression regulation, thus alleviating the oxidative stress driven by inflammatory factors.

Nuclear factor kappa-B (NF-κB) is a family of transcription factors with five members: p65 (RelA), RelB, c-Rel, p105/p50, and p100/p52. The NF-κB pathway is one of the core pathways that regulate inflammatory response and immune homeostasis. Abnormal activation of this pathway may produce a large number of ROS, which is a key factor leading to a variety of malignant diseases (Song et al., 2019; Taniguchi and Karin, 2018; Barreca et al., 2023; Mukherjee et al., 2024). The anti-oxidant stress mechanism of curcumin based on this signaling pathway is illustrated in Figure 3. Yu H. et al. (2020) suggested that the NF-κB pathway includes both classical and non-classical activation modes: (1) The classical NF-κB pathway is fast and transient, and is a key mediator of inflammation and immune response. Under steady-state conditions, p65 and p50 form heterodimers and are fixed in the cytoplasm by NF-κB inhibitors (IκB). Under the stimulation of radiation and pathogens, IκB protein is phosphorylated by IKKs(IκB kinase) and degraded by ubiquitination and proteasome, which promotes the transport of NF-κB dimer to the nucleus and initiates the transcription and expression of pro-inflammatory cytokines, growth factors, chemokines, transcription factors and other genes.(2) The non-classical NF-κB pathway is slow and persistent, and its activation is associated with only a few TNF superfamily receptors. RelB/p52 heterodimer is the key transcription factor of this non-classical pathway and exists in cytoplasm in the form of RelB/p100 under homeostasis. p100 is a precursor of p52 that functions similarly to the IκB protein. Exogenous stimulation causes the accumulation of NIK(NF-κB binding kinase), which co-interacts with IKKα to promote the phosphorylation of p100 and further processing into p52, promoting RelB/p52 heterodimer to enter the nucleus and activate related target genes, thus accelerating the development of multilayer immune cells.

Overexpression of transforming growth factor β-activated kinase-1 (TAK1) and its adaptor TAB1 triggers the activation of NF-κB, which is a key kinase in activating this signaling pathway. Curcumin and its metabolites can reduce the interaction between TAK1 and TAB1, downregulate the phosphorylation levels of TAK1, IKKβ and IκBα, block the ubiquitin-mediated degradation pathway of IκBα, and control the activation of this signaling pathway (Zhang et al., 2018). Curcumin exerts its antioxidant function directly on the NF-κB pathway by inhibiting the degradation of IκBα in the cytoplasm and reducing the phosphorylation level of p65 in the nucleus (Chen et al., 2019), thereby reducing the oxidative stress driven by inflammatory cytokines. Its indirect action is related to the Keap1-Nrf2/ARE signaling pathway. NF-kB activation is inhibited by upregulation of antioxidant gene expression regulated by Nrf2 activation, and this crosstalk mechanism helps cells maintain redox homeostasis (Daverey and Agrawal, 2020).

Structural and functional recovery after myocardial infarction is a key factor in the risk of recurrent cardiovascular events. Moulin et al. (2020) studied the preventive effect of curcumin on myocardial injury caused by chronic intermittent hypoxia (IH). The results showed that the expressions of hypoxia-inducing factor 1α(HIF-1α), $O_{2}^{\cdot -}$ , NF-κBp65 and endoplasmic reticulum stress markers (Grp78 and CHOP) in myocardial tissue of mice exposed to IH were significantly decreased after receiving drug treatment. The infarct size and apoptosis index were significantly decreased after IH induced myocardial ischemia-reperfusion (I/R). Sepsis is an organ dysfunction caused by the host’s dysfunctional response to infection, and its complication septic cardiomyopathy seriously increases the mortality of patients. Chen et al. (2023) used lipopolysaccharide (LPS) to establish sepsis H9C2 cells and rat models to induce myocardial injury. Studies have shown that curcumin therapy downregulates toll-like receptor 1(TLR1)mRNA expression in a dose-dependent manner, inhibits NF-κB phosphorylation by blocking dimerization, effectively downregulates the expression of tumor necrosis factor-α (TNF-α) and interleukin-6 (IL-6), and upregulates the expression of anti-inflammatory factor IL-10. Moreover, the contents of cardiac troponin I(cTnI) and creatine kinase-MB (CK-MB) in serum were significantly decreased, and the cardiac function and survival rate were improved. Chen et al. (2019) established a mouse model of acute gout stimulated by sodium urate (MSU) to study the therapeutic effect of curcumin on acute gout induced by MSU deposition. The results showed that curcumin could inhibit the degradation of IκBα protein in THP-1 cells, control the expression levels of p-p65, p-p50, p65, p50, and block the activation of NF-κB pathway. In addition, the mRNA expressions of inflammatory cytokines IL-1β, IL-6, TNF-a and cyclooxygenase-2 (COX-2) were downregulated in a dose-dependent manner, ROS accumulation in mitochondria was significantly reduced, and the swelling symptoms of ankle joint and foot ball of model mice were alleviated. In the LPS-induced bovine mammary epithelial cell (MAC-T) inflammation model (Li et al., 2021), 10 µM curcumin significantly reduced ROS and MDA accumulation in the 100 µg/mL induced model, increased the viability of MAC-T cells in a dose-dependent manner, enhanced the activity of T-SOD and GSH, inhibited the levels of NF-κB subunits p65 and p50, downregulated the expression of IL-8, IL-1β, IL-6 and TNF-a, and alleviated the cell membrane damage caused by LPS. Cypermethrin (Cyp) is hydrophobic and can easily disturb the cell membrane structure to induce liver injury. Nano-Liposomes Double Loaded with Curcumin significantly enhances SOD,CAT activities and GSH levels in rats, and the high levels of expression of oxidative stress markers (4-HNE), inflammatory markers (NF-κB) and apoptotic markers (Bax and Apaf-1) under Cyp were successfully reversed, thus increasing cell membrane permeability and significantly improving CyP-induced liver dysfunction (Hussain et al., 2023). The organic pesticide malathion tends to cause an increase in acetylcholine, leading to a variety of clinical diseases including renal dysfunction. Curcumin administration significantly decreased serum creatinine concentration, blocked the activation of NF-κB pathway by regulating the endogenous antioxidant oxidase defense system (HO-1, TAC and GSH), by inhibiting the degradation of IκBα and the nuclear translocation of NF-κB, decreased the expression levels of IL-1, IL-6 and TNF-a, and protects renal tissue from malathion induced oxidative stress (Eldesoqui et al., 2023).

## 4 NADPH oxidase signaling pathway

Nicotinamide adenine dinucleotide phosphate oxidase (NOX) and mitochondrial electron transport chain is the main source of ROS. The former catalyzes the reduction of O_2_ to $O_{2}^{\cdot -}$ or H_2_O_2_ by transmembrane electron transfer and is coupled to the oxidation of NADPH (Vermot et al., 2021). The enzyme was first discovered on the surface of phagocytes and is a multi-subunit complex enzyme. Its catalytic core Nox2 (also known as gp91^phox^) and p22^phox^ are stable in the form of heterodimer on the cell membrane. The phosphorylation signal of cytoplasmic subunits (p47^phox^, p67^phox^, p40^phox^) promotes their aggregation to the Nox2 membrane binding site, and recruits Rac-GDP isolated in the cytoplasm. Rac-GTP can be generated after conversion, and finally the connection with p67^phox^ leads to the activation of Nox2. There are seven types of NOX in mammals (NOX1-5, DUOX1-2), all of which contain a six-way transmembrane helical domain (TM) and cytoplasmic dehydrogenase domain (DH). The TM domain chelates two heme groups to form an electron channel within the cell membrane. The DH domain consists of an N-terminal lobe that binds the FAD cofactor and a C-terminal NADPH-binding lobe (Ogboo et al., 2022). Its transmembrane electron transfer function depends on the reducibility of NADPH, which provides two electrons to FAD to reduce it to FADH2. The first electron is quickly transferred from the proximal heme of NOX to the distal heme, and then to O_2_ to form $O_{2}^{\cdot -}$ . The second electron carried by semi-quinone FAD is transferred in the same way, promoting ROS production.

**FIGURE 4:**
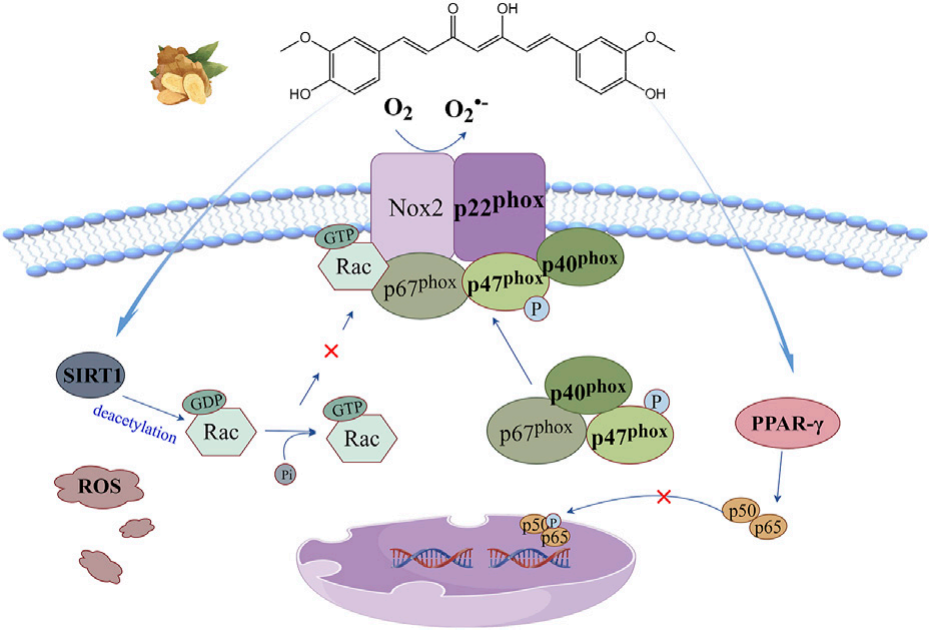
NADPH oxidase signaling pathway: curcumin can enhance PPAR-γ activity to inhibit hypoxia-induced NF-κB nuclear translocation, or promote SIRT1 expression to reduce Rac1 binding potential to p67phox, thereby blocking this signaling pathway.

The anti-oxidant stress mechanism of curcumin based on this signaling pathway is illustrated in Figure 4. It is generally believed that curcumin regulates this signaling pathway by two mechanisms. Curcumin, as a natural agonist of peroxisome proliferator-activated receptor-γ (PPAR-γ), inhibits hypoxia-induced NF-κB nuclear translocation by enhancing PPAR-γ activity and indirectly controls NF-κB-driven cytoplasmic subunit transcription to regulate this signaling pathway (Wu et al., 2016). Silencing information regulatory factor 1(SIRT1) is an NAD^+^ dependent histone deacetylase, which can deacetylate Rac1 and significantly reduce the binding potential of Rac1 and p67^phox^ (Xia et al., 2021). Curcumin has the ability to promote the expression of SIRT1, thereby blocking this signaling pathway (Feng et al., 2019).

Bioaccumulation of lead and cadmium in the food chain is potentially harmful to human health and can significantly increase the risk of cardiovascular disease. Tubsakul et al. (2021) continuously exposed SD male rats to low levels of lead acetate, cadmium chloride or their combination in drinking water to build a hypertensive rat model induced by heavy metal exposure, which showed increased intravasal superoxide formation and significant increases in systolic and diastolic blood pressure. It was found that curcumin therapy reversed the upregulation of gp91^phox^ expression under metal exposure, partially prevented the increase of MDA and protein carbonyl levels, increased intracellular GSH levels and their redox ratio, significantly alleviated oxidative stress responses, and reduced blood pressure. Abnormal proliferation and migration of vascular smooth muscle cells (VSMCs) is a key factor in the initiation of atherosclerosis and hypertension. Panthiya et al. (2023a) studied the effects of curcumin metabolites on angiotensin II (AngII)-induced proliferation and migration of aortic VSMCs in rats. The results showed that pretreatment with hexahydrocurcumin significantly reversed the upregulation of NOX1 and NOX4 expression in AngII-induced state, enhanced the activity of PPAR-γ and its coactivator 1α(PGC-1α), downregulated cell cycle regulatory protein (cyclinD1), enhanced the expression of p21, inhibited NOX-mediated ROS production and the phenotypic transformation and abnormal proliferation of VSMCs, suggesting the potential of curcumin in the prevention and treatment of arteriosclerosis. Zhai et al. (2015) explored the protective function of curcumin against LPS-induced VSMCs injury based on TLR4/NOX/ROS signaling pathway and the release of inflammatory factors. The results showed that curcumin pretreatment could effectively control the overexpression of TLR4 and the activation of downstream NOX signaling pathway, significantly inhibit the upregulated expression of p22^phox^ mRNA and the increased secretion of TNF-α and IL-1 under the induction of LPS, thus alleviating the oxidative stress and inflammation caused by endotoxin. Gupta et al. (2021) established a human intestinal cell line injury model induced by wheat gluten. Treatment with olysin and H_2_O_2_ significantly increased the expression of p47^phox^ and stressed the transfer of NF-κB protein from cytoplasm to nucleus. Curcumin pretreatment can reduce NOX activity by about 34%, block nuclear translocation of NF-κB, inhibit transcription and translation levels of multiple pro-inflammatory cytokines, and maintain cellular redox and immune homeostasis. Li et al. (2017) exposed H9C2 embryonic rat cardiogenic cells to 0.2 mM palmitate (PA) medium. Nano curcumin treatment significantly inhibited the increase of Rac1 activity and the expression of p22^phox^, p47^phox^, p67^phox^ and gp91^phox^ induced by PA exposure. The intracellular ROS levels and lipid peroxidation were strongly decreased, and the decrease of Bcl-2/Bax ratio in PA state was completely reversed, alleviating myocardial apoptosis and myocardial injury lipid toxicity. Sharma and Nehru (2018) injected LPS into the substantia nigra of rats to establish an animal model of Parkinson’s disease. Curcumin treatment significantly reversed the upregulation of p47^phox^, p67^phox^ and gp91^phox^ in the pathological state, significantly reduced the activity of NF-κB protein and the expression of pro-inflammatory cytokines, and increased the level of GSH and its redox ratio. Moreover, it inhibits the aggregation of α-synuclein in dopaminergic neurons and exerts its antioxidant and neuroprotective functions.

## 5 MAPK signaling pathway

Mitogen-activated protein kinase (MAPK) belong to the family of serine/threonine kinase, mainly including extracellular signal-regulated kinases (ERK1/2), p38 kinase, c-Jun N-terminal kinase(JNK) and ERK5 four family. Each pathway branch consists of at least three cascades and involves three core kinases: MAPKKK(MAPK kinase kinase), MAPK (MAPK kinase) and MAPK. The basic mode of this signaling pathway is to release signaling factors to phosphorylate the three core kinases successively, thereby regulating the expression of downstream transcription factors and cell behavior (Leyane et al., 2021).

**FIGURE 5:**
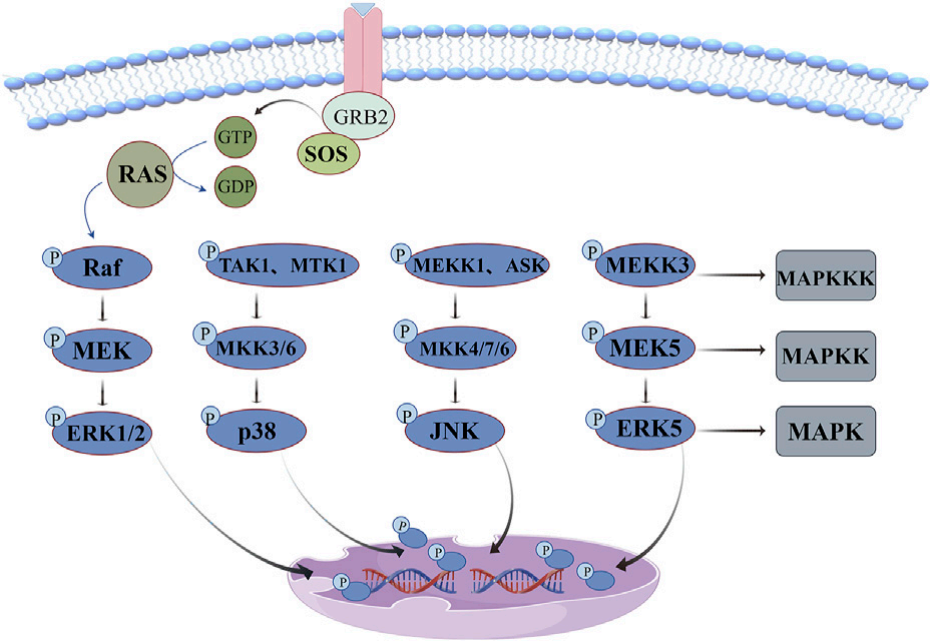
MAPK signaling pathway: ERK pathway dysfunction is very common in cancer, p38 kinase and JNK pathway are often activated by environmental stress and inflammatory factors, and ERK5 pathway plays an important role in regulating cell proliferation and differentiation.

The regulatory network of the MAPK signaling pathway is illustrated in Figure 5. ERK signaling pathway, also known as the classical pathway, is involved in cell proliferation, apoptosis, invasion and migration, and its dysfunction is very common in cancer. This pathway gradually activates RAF kinase to initiate signaling cascade through growth factor binding to cell surface receptors, recruitment of SOS to activate RAS protein and other pathways, which causes serine phosphorylation of MEK1 or MEK2, resulting in tyrosine and threonine (Tyr, Thr) of ERK protein phosphorylation and transfer into the nucleus. Ultimately, phosphorylation of multiple transcription factors regulates gene expression (Kciuk et al., 2022). Unlike the ERK pathway, p38 kinase and JNK pathways are poorly responsive to mitogen and are usually activated by environmental stress and inflammatory factors (Canovas and Nebreda, 2021). p38 kinases exist in four subtypes: P38α, p38β, p38γ, and p38δ, which have high amino acid homology but differ in downstream targets. p38α has the highest expression level (except in some brain regions) and is the most widely studied. p38/MAPK is almost ubiquitous throughout the body and can be activated by cellular stress such as radiation, cellular inflammatory factors, osmosis, and heat shock (Leyane et al., 2021). There are dozens of MAPKKK(TAK1, MTK1, etc.) participating in its upstream cascade. MAPKK(MKK3/6) is highly specific to p38 kinase and activates Thr and Tyr double phosphorylation in the ring (Thr180 and Tyr182 in P38α) (Canovas and Nebreda, 2021). The activation of the JNK pathway also depends on the expression of MAPKKK(MEKK1, ASK), inducing the activation of MAPKK(MKK4/7/6) and ultimately promoting the dual phosphorylation of Thr183 and Tyr18 in the ring (Cicenas et al., 2017). ERK5, which has been less studied, has the same Thr-Glu-Tyr activation sequence as ERK1/2. The core kinases in this pathway mainly include MAPKKK(MEKK3) and MAPKK(MEK5), which play an important role in regulating cell proliferation and differentiation (Cicenas et al., 2017; Tubita et al., 2020).

Vascular remodeling in hypertensive patients involves a variety of complex mechanisms such as vascular wall thickening, oxidative stress and fibrosis, which can seriously increase the risk of stroke and cardiovascular disease. Panthiya et al. (2022b) used nitric oxide synthase inhibitor (L-NAME) to construct a hypertensive rat model, which showed an increase in main artery wall thickness, cross-sectional area and collagen deposition, accompanied by an increase in markers of oxidative stress. Studies have shown that hexahydrocurcumin treatment significantly enhances the activities of eNOS, p-eNOS and SOD, inhibits the expression of p-ERK1/2, p-JNK and p-p38 proteins, significantly reduces the production of vascular ROS and plasma MDA levels, and weakens vascular hypertrophy and remodeling mediated by MAPK activation. Moreover, by inhibiting the expression of fibrinogene-related proteins, the vascular fibrosis of rats was alleviated, the phenotypic transformation of VSMCs in aortic tissue was inhibited, and the vascular dysfunction and vascular remodeling were effectively improved, providing a theoretical basis for curcumin as a treatment drug for hypertension. Oxidative damage caused by myocardial reperfusion is an important cause of morbidity and death in patients with ischemic cardiomyopathy. Wei et al. (2019) established the hypoxia/reoxidation (H/R) injury model of H9c2 cells, and the study showed that curcumin significantly reversed the increase of lactate dehydrogenase (LDH) and MDA levels and the decrease of SOD activity under the injury condition, significantly downregulated the expression levels of p-ERK1/2, p-JNK and endoplasmic reticulum stress markers (GRP78, CHOP), effectively alleviated H/R-induced ROS accumulation, endoplasmic reticulum stress and cell apoptosis, providing a basis for the clinical application of curcumin in cardiac ischemia-reperfusion injury. Exposure to high concentrations of copper can cause cardiotoxicity and significantly increase the incidence of cardiovascular disease in humans. Sarawi et al. (2021) used rat gavage of CuSO_4_ to construct a heart injury model. The study showed that curcumin and nanocurcumin treatment effectively enhanced the activities of GSH, SOD and CAT, decreased the phosphorylation levels of p38MAPK, JNK and ERK1/2, upregulated anti-apoptotic factors Bcl-2 and BAG-1, and inhibited Bax and caspase-3. The levels of cTnI, CK-MB, LDH, MDA and pro-inflammatory mediators in rat myocardium were significantly decreased, and the heavy metal-induced apoptosis of cardiomyocytes was protected by regulating oxidative stress and inflammatory response. Buccarello et al. (2020) explored the modification effect of curcumin on the acylation of small ubiquitin-like modifier (SUMO) in the H_2_O_2_-induced oxidative stress model of SHSY5Y cells and the cross-talk mechanism between SUMO-1 and JNK. The results showed that low concentration of curcumin (5 μM) could control the phosphorylation of SUMO-1 at normal level, significantly reduce the phosphorylation levels of JNK and ERK proteins, reduce the release of LDH, and completely counteract the toxic side effects of 0.5 mM H_2_O_2_ induced oxidative stress. Moreover, H_2_O_2_ stimulation induces SUMO-1 accumulation in nucleosomes, recruiting p-JNK from cytoplasm to intracellular clusters and perinuclear. This co-localization effect of P-JNK-SUMO-1, which causes abnormal protein aggregation, can be destroyed by curcumin, preventing cells from initiating the death cascade and preventing cell disorders caused by oxidative stress. Fallahnezhad et al. (2023) showed that curcumin nanoparticles reversed the increased levels of ROS, MDA, TNF-α, IL-6 and IL-1β in hippocampus and cortex of rats treated with BPA, significantly upregulated the expression of p-AKT and p-ERK1/2 in tissues, and downregulated the expression of p-P38 and p-JNK. Moreover, the increased expression of glutamate receptors and memory-related proteins may have a potential protective effect on subacute neurotoxicity and learning and memory impairment of BPA in hippocampus and cortex of rats.

## 6 PI3K/Akt signaling pathway

Phosphatidylinositol3-kinase/protein kinase B (PI3K/Akt) signaling pathway plays an important role in the recruitment of inflammatory factors and angiogenesis, and is one of the core pathways regulating cell proliferation, metabolism, movement and survival. Three classes of PI3K have been identified: Class I, Class II and Class III. In addition to the common substrate Akt, each kinase class has specific substrates and effectors. Class I PI3K is the most widely studied heterodimer composed of catalytic subunit p110 and regulatory subunit P85. The binding of a cytokine or growth factor to the corresponding receptor causes autophosphorylation of the tyrosine, which recruits PI3K to the cell membrane for activation, and its catalytic subunit p110 induces phosphorylation of the phosphatidylinositol 4,5-bisphosphate (PIP2), converting it to phosphatidylinositol 3,4,5-triphosphate (PIP3). PIP3 acts as a second messenger to recruit Akt and 3-phosphoinositide-dependent kinase 1 (PDK1), and allows PDK1 to phosphorylate Akt’s kinase domain (Thr308). After its regulatory domain (Ser473) is activated by mTORC2, the enzyme activity can be fully released (Wong et al., 2021; He et al., 2021). This pathway has crosstalk with multiple signal networks such as MAPK and NF-κB, and inhibition of key molecular targets can control the aggregation of inflammatory cells to the inflammatory site and alleviate the resulting oxidative stress response (He et al., 2021).

**FIGURE 6:**
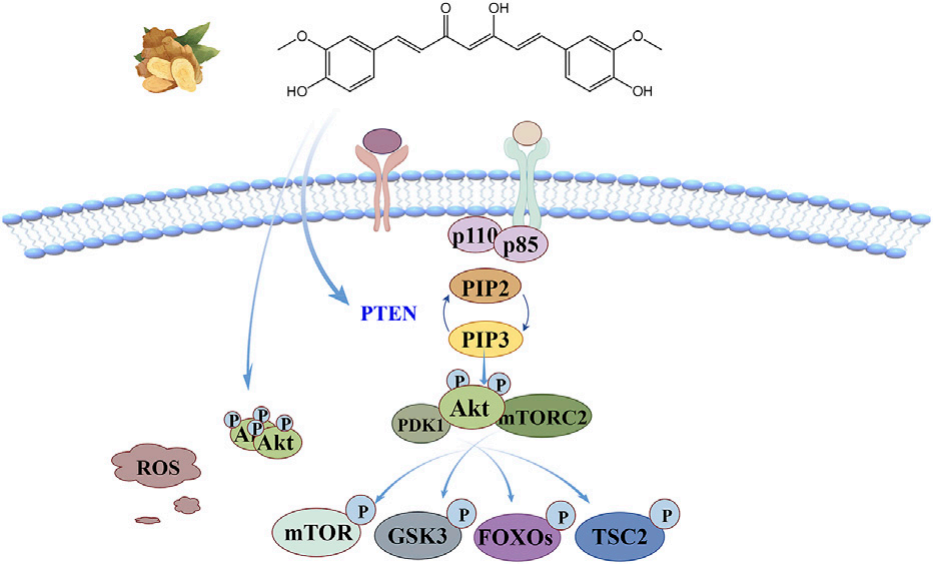
PI3K/Akt signaling pathway: curcumin not only binds tightly to Akt1 by binding the four hydrogen bonds in the pocket, but also negatively regulates this signaling pathway through PTEN, which inhibits downstream kinase activation by dephosphorylation of PIP3 to PIP2.

The anti-oxidant stress mechanism of curcumin based on this signaling pathway is illustrated in Figure 6. According to molecular docking model analysis (Zhang et al., 2023), curcumin tightly binds Akt1 through four hydrogen bonds in the binding pocket, controlling the phosphorylation level of Akt. Curcumin can also negatively regulates this signaling pathway through phosphatase and tensin homology (PTEN), which inhibits downstream kinase activation by dephosphorylation of PIP3 to PIP2 (Zhuang et al., 2019). There are more than 100 Akt substrates, mainly including mammalian target of rapamycin (mTOR), multifunctional serine and threonine protein kinase glycogen synthase kinase 3(GSK3), forkhead transcription factor (FOXOs), tuberous sclerosis Complex 2(TSC2), etc. Akt phosphorylates Thr and Ser in a sequentially dependent manner, thus activating downstream effectors (He et al., 2021; Mossmann et al., 2018).

Coronary artery recirculation is an effective treatment for ischemic heart disease, but blood perfusion of ischemic myocardium may further aggravate the damage of myocardial structure and function. Wu et al. (2021) constructed a rat model of myocardial ischemia-reperfusion injury (MIRI) to explore the molecular mechanism of the protective effect of curcumin on myocardium based on PI3K/Akt/mTOR signaling pathway. The results showed that curcumin could significantly reduce the myocardial infarction area, upregulate the expression of mTOR, PI3K and p-Akt, increase the expression of Bcl-2/Bax, decrease the level of autophagy, significantly increase the activity of GSH and SOD, and decrease the level of serum marker enzymes CK-MB, LDH and MDA. MIRI can be effectively combated by enhancing myocardial antioxidant capacity and inhibiting myocardial cell apoptosis. Doxorubicin (DOX) is one of the most effective antitumor agents, but long-term high doses can induce oxidative stress and pyroptosis of cardiomyocytes, which can seriously increase the risk of left ventricular dysfunction, dilated cardiomyopathy, and even congestive heart failure. Yu W. et al. (2020) used DOX to build a mouse heart injury model. Studies showed that curcumin treatment significantly enhanced SOD activity, reduced $O_{2}^{\cdot -}$ and MDA levels, and offset the DOX-induced decline in AKT and mTOR phosphorylation levels, partially reversing the NLRP3 inflammator-mediated changes in pyrogenic protein markers. By enhancing antioxidant capacity and inhibiting autophagy and pyrodeath of cardiomyocytes, the dysfunction of cardiomyocytes was effectively alleviated. Oxidative stress, apoptosis and autophagy are the key molecular mechanisms of MIRI after acute myocardial infarction. Chen et al. (2021) explored the cardioprotective effects of tetrahydrocurcumin *in vivo* and *in vitro* based on the PI3K/AKT/mTOR pathway. The results showed that tetrahydrocurcumin treatment significantly reversed the H/R-induced increase in Bax/Bcl-2 expression, enhanced intracellular SOD and CAT activities, decreased the ratio of LC3-II/LC3-I in cardiomyocytes, downregulated the expression of Beclin-1, effectively enhanced the antioxidant capacity of cells and inhibited the apoptosis and autophagy events of cardiomyocytes. The reversal of these functions by PI3K inhibitors and mTOR inhibitors further confirms the key role of this signaling pathway in the cardiac protection of tetrahydrocurcumin. Abuelezz et al. (2020) induced a rat model of polycystic ovary syndrome with letrozole, resulting in significant disturbance of oxidative stress markers, sex hormone levels and dyslipidemia in the body. Enzyme-linked immunosorbent assay (ELISA) and pancreatic tissue homogenate detection showed that nanomorcumin alleviated insulin resistance and pancreatic functional deficit in a dose-dependent manner. PI3K/AKT/mTOR were successfully restored to normal levels, MDA levels were significantly decreased, GSH and SOD activities were increased, and oxidative stress damage induced by letrozole was significantly improved. Abnormal methylation of the PTEN promoter causes cell fibrosis, resulting in joint contracture. Zhuang et al. (2019) believe that curcumin can effectively promote the demethylation of PTEN and upregulate its expression, significantly control its phosphorylation level, further inhibit the PI3K/Akt signaling pathway, and impede the proliferation and migration of myofibroblasts.

## 7 AMPK signaling pathway

Amp-activated protein kinase (AMPK) is a core controller that regulates cellular energy homeostasis, and a large change in ATP/AMP ratio will promote its activation. The energy balance between anabolism and catabolism is maintained by regulating the phosphorylation of key proteins in lipid homeostasis, glycolysis and mitochondrial homeostasis. AMPK is a heterotrimeric complex composed of catalytic α-subunits and regulatory β, γ-subunits. The α-subunit contains a key kinase domain (KD) in which thr172 can be phosphorylated by liver kinase B1(LKB1), calcium/calmodulin-dependent kinase kinase 2 (CaMKK2) and related upstream kinases, and this is necessary to stimulate AMPK activity. The carbohydrate binding module (CBM) of the β-subunit can be linked to glycogen, and it has four tandem cystathione-β-synthase (CBS) domains, which can directly bind AMP or ATP, so it has the ability to rapidly respond to changes in the ATP/AMP ratio in tissues, and can lead to allosteric activation of phosphorylated AMPK after binding (Herzig and Shaw, 2018). The interface between KD and CBM forms a unique interaction pocket, which has become a major binding site for many small molecule activators, which is independent of the activation potential of AMPK outside of LKB1, CaMKK2 and upstream kinases (Trefts and Shaw, 2021).

**FIGURE 7:**
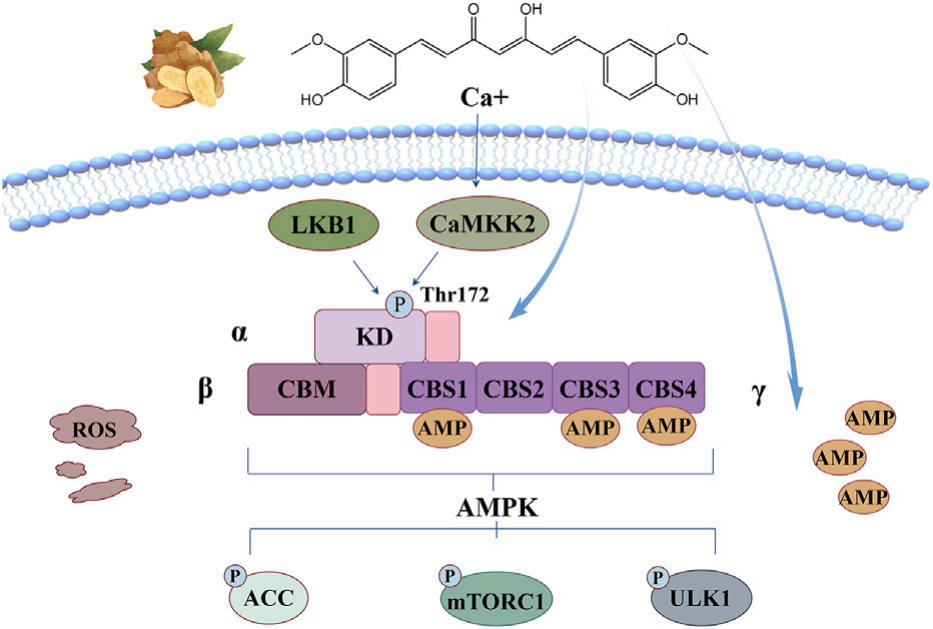
AMPK signaling pathway: curcumin can bind directly to AMPK at its allosteric regulatory site through hydrogen bonding and π-π stacking interactions to form a stable CUR-AMPK complex that promotes allosteric activation or activates it by increasing cAMP levels.

The anti-oxidant stress mechanism of curcumin based on this signaling pathway is illustrated in Figure 7. Curcumin has been reported to bind directly to AMPK at its allosteric regulatory site through hydrogen bonding and π-π stacking interactions to form a stable CUR-AMPK complex that promotes allosteric activation (Liu et al., 2017) or activates it by increasing cAMP levels (Iside et al., 2020). Activated AMPK functions primarily by phosphorylating specific downstream signals. Firstly, Ser79 of acetyl-CoA carboxylase (ACC) can be phosphorylated to inhibit the biosynthesis of cholesterol and fatty acids, which also has an inhibitory effect on ferroptosis (Trefts and Shaw, 2021). The second classic function is achieved by blocking mTORC1 activation. AMPK inhibits the activity of Ras homolog (Rheb) by directly phosphorylating Ser residues of Raptor, a key scaffold protein, or by promoting the formation of complexes between TSC2 and TSC1, thereby preventing mTORC1 from recruiting substrates to regulate cell metabolism (Peng et al., 2022). The third classical function is the activation of autophagy. AMPK regulates the catalytic activity of UNC-51-like autophagy activating kinase 1(ULK1) by directly phosphorylating multiple sites and inhibiting the inhibition of ULK1 by mTORC1, thereby promoting the initiation and execution of autophagy (Trefts and Shaw, 2021).

Lipotoxicity is an important cause of clinical heart disease, which may be closely related to lipid deposition and ROS accumulation in cardiac tissue (Zhang et al., 2019). H9C2 cells were co-incubated with PA to construct a lipopotoxic cardiocyte injury model, and the study showed that the intervention of nano-curcumin significantly inhibited ROS levels in the injured cells, and restored MDA content and SOD activity to normal levels. The decreased level of p-AMPK and the increased levels of downstream targets p-mTORC1 and p-p70S6K in PA-induced state were significantly reversed, which greatly reduced the ratio of Bcl-2/Bax, alleviated myocardial oxidative stress and apoptosis, suggesting the potential of curcumin in preventing myocardial lipid toxic injury. Restoring endothelial cell function is an effective treatment strategy for atherosclerosis. Zhao et al. (2021) studied the protective mechanism of curcumin in lipotoxic injury of human umbilical vein endothelial cells (HUVECs) induced by oxidized low-density lipoprotein (ox-LDL). The results showed that curcumin treatment significantly restored cell viability, enhanced the ability of cell to form independent tubular structures and migration, reduced ROS levels and the release of inflammatory factors, reversed the decline of p-AMPK and the increase of p-mTOR and p-p70S6K in ox-LDL -induced state, and upregulated the expression of LC3-II protein. Thus, autophagy flux was restored and lipid toxicity of endothelial cells was reduced. Kong et al. (2020) constructed two BNLCL.2 cell models *in vitro*: normal autophagy model (siCTR) and autophagy deficiency model (siBECN1). It was found that curcumin can restore the expression of peroxisome proliferator-activated receptor-α (PPAR-α) to the physiological level, upregulate AMPK mRNA expression, downregulate mTOR mRNA expression, enhance GSH activity in a dose-dependent manner, and significantly reduce ROS levels and oxidative stress in hepatocytes. This may be achieved by activating the upstream autophagy signal of AMPK/PI3K/AKT/mTOR mediated by PPAR-α to enhance autophagy and inhibit the EMT process, providing a new idea for the treatment of liver fibrosis. Sadek et al. (2024) induced experimental autoimmune encephalomyelitis (EAE) in mice with spinal cord homogenate (SCH) and Freund’s adjuvant, and simulated multiple sclerosis to evaluate the therapeutic effect of curcumin on EAE-induced autonomic, motor, cognitive, and sensory disorders. The results showed that curcumin intervention significantly increased the levels of p-AMPK Thr172 and SIRT1, alleviating neuronal demyelination and death induced by EAE. In addition, the neuroprotective axis AMPK/SIRT1 can activate Nrf2, enhance the activity of endogenous antioxidant enzymes to repair EAE induced oxidative stress, and significantly reduce the hippocampal tissue changes in mice. Tanaka et al. (2023) knocked out AMPKα1 gene to analyze whether AMPK regulated osteoclast differentiation through oxidative stress and maintained the dynamic balance between osteoclast bone resorption and osteoblast bone formation. It was found that in RAW264.7 cells that silted AMPK gene, the expression of inflammatory genes was upregulated, resulting in the expression defects of Nrf2 and HO-1. Moreover, NF-κB ligand (RANKL) induced osteoclast differentiation and osteoclast gene expression were significantly enhanced, bone mass decreased. Curcumin treatment significantly improved oxidative stress and negatively regulated the expression of osteoclast genes to protect bone loss.

## 8 Wnt/β-catenin signaling pathway

Wnt proteins are secreted glycoproteins rich in cysteine, and their biological activity depends on the protein acylation modification of porcupine O-acyltransferase PORCN (Nile and Hannoush, 2016). At rest, β-catenin in the cytoplasm is isolated in a “destruction complex” co-formed with AXIN, Adenomatous Polyposis Coli (APC), casein kinase 1α(CK1α), and GSK3β. CK1α and GSK3β phosphorylate respectively Ser45 and Ser33, 37 and Thr41 of β-Catenin, and rely on ubiquitination and proteasomal degradation pathways to maintain them at low levels. It also allows transcription co-suppressor (GROUCHO protein) to bind to T-cell factor/lymphoid enhancer factor (TCF/LEF) in the nucleus, thereby blocking the transcription of Wnt target genes (Russell and Monga, 2018). Stimulated by oxidative stress sources, ROS accumulates and promotes the production and release of Wnt proteins through multiple pathways (Vallée and Lecarpentier, 2018a; Vallée et al., 2019b). Wnt proteins bind to the frizzled receptor (Fzd) and low-density lipoprotein receptor-related proteins 5 and 6 (LRP5/6) on the receiving cell membrane and causes the phosphorylation of the latter, inducing the recruitment of signal transducers (DVL) and AXIN to this site, thereby inhibiting the activity of GSK3β and blocking the phosphorylation and degradation pathway of β-Catenin. It causes the cytoplasmic accumulation of β-Catenin and promotes its nuclear translocation. Subsequently, β-Catenin binds to TCF/LEF to activate Wnt target genes, thus regulating various biological processes such as cell proliferation, differentiation and migration. It can induce cell proliferation and at the same time give specific growth tissue shape, indicating the orderly growth of new cells and complete distribution (Rim et al., 2022; Nusse and Clevers, 2017).

**FIGURE 8:**
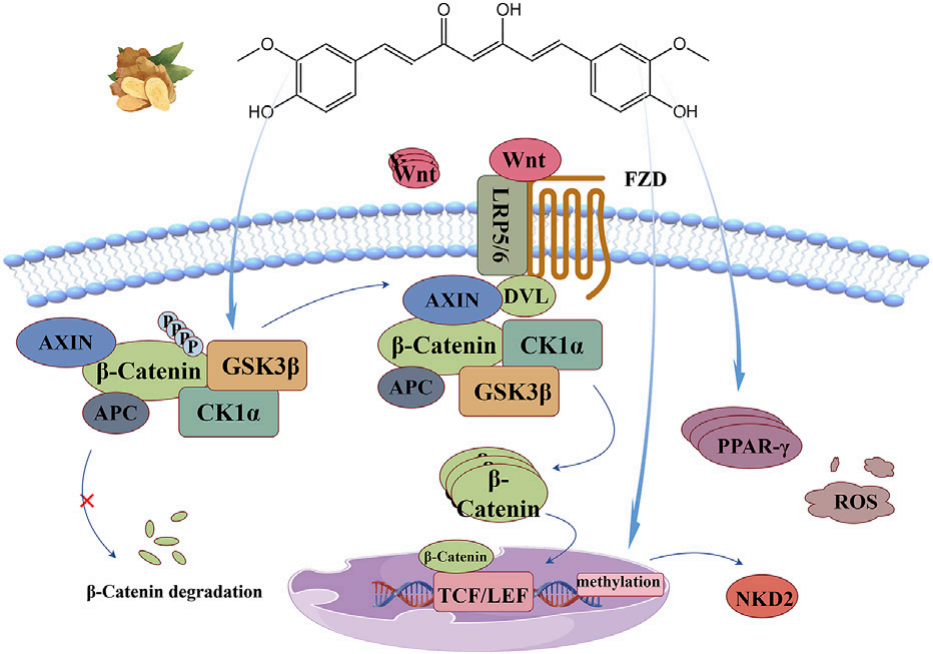
Wnt/β-Catenin signaling pathway: Curcumin directly stimulates GSK3β activity, uses the negative feedback relationship between PPAR-γ and Wnt/β-Catenin pathway, or induces DNA demethylation to upregulate NKD2 expression, thus negatively regulating this signaling pathway.

The anti-oxidant stress mechanism of curcumin based on this signaling pathway is illustrated in Figure 8. Curcumin, as a natural agonist of PPAR-γ, uses the negative feedback relationship between PPAR-γ and Wnt/β-Catenin pathway (Vallée and Lecarpentier, 2018a), or directly stimulates GSK3β activity to disrupt the nuclear translocation of β-Catenin (Wang et al., 2018; He et al., 2014) and inhibit this signaling pathway. Methylation of the gene promoter region leads to the stable silencing of naked cuticle homolog 2 (NKD2), resulting in abnormal activation of the Wnt signaling pathway. It has been reported that curcumin can induce DNA demethylation to upregulate the expression of NKD2 and negatively regulate this signaling pathway (Zhu et al., 2024).

Zhu et al. (2024) established an I/R model of superior mesenteric artery occlusion in SD rats to explore the intestinal protective effect of curcumin based on Wnt/β-Catenin signaling. The results showed that curcumin could reverse the increase of β-Catenin and p-GSK3β, significantly increase the activity of SOD and GSH-Px and decrease the content of MDA. The experiment of transfecting Caco-2 cells with oe-NKD2 loaded by lentivirus before H/R injury confirmed that curcumin upregulated the expression of NKD2 through DNA demethylation to inhibit the signaling pathway and play a protective role in intestinal tract. Wang et al. (2018) cultured non-small cell lung cancer (NSCLC) cell line A549, and found that curcumin intervention could reduce the ROS level of A549 cells and increase the activities of SOD and γ-glutamylcysteine synthetase (γ-GCS). Moreover, curcumin strongly inhibited the expression of β-Catenin, p-GSK3β and its downstream targets cyclinD1 and c-Myc, decreased cell viability in a dose-dependent manner, and controlled NSCLC proliferation induced by upregulation of Wnt/β-Catenin pathway mediated by oxidative stress. Zhao et al. (2022) pretreated rat osteoblasts with curcumin with a concentration gradient, and established a oxidative stress model induced by 50 μmol/L H_2_O_2_ to explore the mechanism of curcumin’s protective function of osteoblasts. Studies have shown that curcumin pretreatment can increase the activity of SOD, CAT and T-AOC and decrease the content of MDA in cells, reverse the down expression of Wnt5a and β-Catenin induced by H_2_O_2_, significantly upregulate the expression of alkaline phosphatase (ALP), promote the formation of calcium mineralization nodules, and alleviate the inhibition of osteogenesis under oxidative stress. Ho et al. (2016) found that curcumin pretreatment significantly restored the inhibitory effect of high glucose on the expression of Wnt5a and nuclear β-catenin protein, inhibited the increased expression reversed the increase of β-Catenin and p-GSK3βof transforming growth factor (TGF-β1) and fibronectin, and downregulated the expression of 8-OHDG. Improve diabetic glomerular injury by repairing superoxid-mediated Wnt5a/β-catenin signaling pathway and alleviating oxidative stress.

## 9 Notch signaling pathway

Notch receptors are large single-pass type I transmembrane protein that mediates signal transduction of extracellular ligand and is mainly involved in immune regulation, cell proliferation, differentiation, apoptosis and other biological processes. There are four Notch analogues in mammals, each with four cutting sites. In Golgi, Furin-like convertase attacks the S1 site of the protein, transforming its polypeptide structure into heterodimer structures linked by non-covalent interaction, forming mature Notch receptors in the ligand-free state, and being transported to the surface of the cell membrane (Kopan and Ilagan, 2009). The cell surface receptor (Notch1-4) effectively binds to the DSL ligands (Jag1, Jag2 and triangle ligands Dll1, Dll3, Dll4, etc.) present on the surface of adjacent cells after 11–12 repeated trans-interactions, changing the cis-conformation of the receptor and exposing the S2 cleavage site. This site is attacked by a disintegrin and metalloproteases (ADAM), which divides the Notch receptor into extracellular, transmembrane, and intracellular domains. Ligand-expressing cells take the former away through endocytosis. And the latter continues to be attacked at the S3 site by a γ-secretase complex, which cleaves multiple cleavable bonds and continues to split towards the S4 site until the β-polypeptide escapes the phospholipid bilayer and releases the intracellular domain (NICD) structure into the nucleus (Kopan and Ilagan, 2009; Kovall et al., 2017; Meurette and Mehlen, 2018). NICD does not directly bind to DNA to affect gene transcription, but competes with ubiquitous corepressor (Co-R) proteins and histone deacetylases (HDACs) for DNA-binding protein CSL to be released from the complex. The interaction between NICD and CSL induces the recruitment of transcription coactivators (Co-A, MAML, Co-As, etc.) to this site to regulate the transcription of corresponding target genes (Meurette and Mehlen, 2018).

**FIGURE 9:**
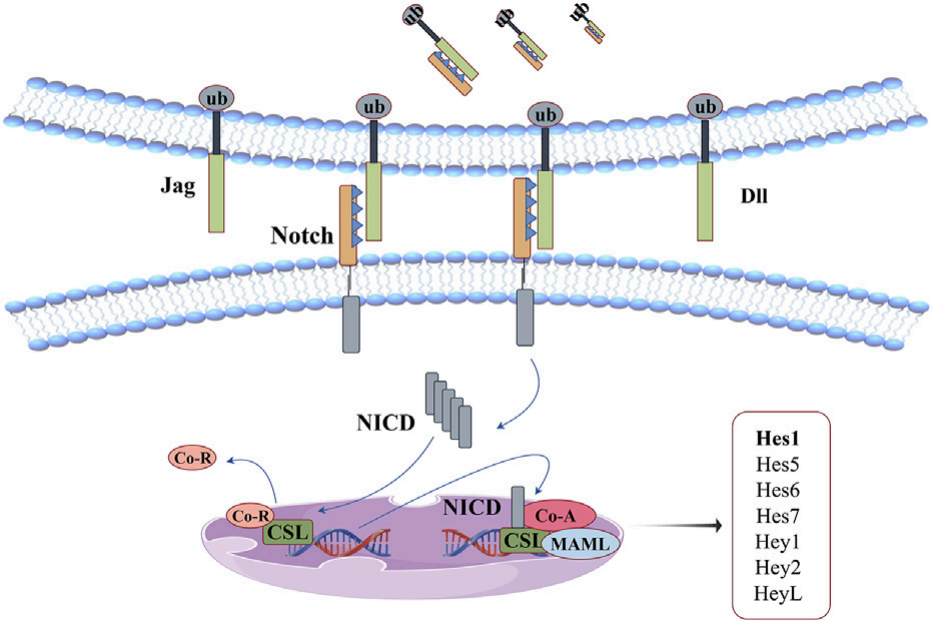
Notch signaling pathway: curcumin can bind Notch-1 protein and the bHLH domain of Hes1. This interaction helps curcumin effectively control this signaling pathway.

The function of this signaling pathway in anti-oxidative stress has been confirmed in several studies (Xuan et al., 2020; Luo et al., 2021; Song C. et al., 2022). Its mechanism is illustrated in Figure 9. In addition, molecular docking studies have shown (Tandon et al., 2020) that curcumin can bind Notch-1 protein through 7 hydrogen bonding interactions, and form 7 electrostatic interactions with its active site residues, and bind to the bHLH domain of Hes1 through 2 hydrogen bonding interactions, 11 electrostatic interactions and 2 hydrophobic interactions. This interaction helps curcumin effectively control this signaling pathway. Among them, hair-division related enhancers (Hes1, Hes5, Hes6, Hes7, and Hey1, Hey2, HeyL) are key downstream targets of the Notch signaling pathway. They have high structural homology and share the same bHLH domain, allowing homodimerization or heterodimerization of both or other transcriptional regulators containing this domain, which determines their specificity in binding to DNA (Zanotti and Canalis, 2016).

Gao et al. (2019) used different concentrations of H_2_O_2_ to construct astrocyte spinal cord injury models, and the contents of ROS, IL-6 and TNF-α in the cells showed abnormal increases. Compared with H_2_O_2_ group, curcumin treatment group significantly decreased the expression of Notch1, Hes1 and Bax proteins, significantly decreased ROS levels and inhibited astrocyte apoptosis. Its protective effect on spinal cord injury may be achieved by down-regulating Notch pathway to relieve oxidative stress and inflammatory response. Zhu et al. (2019) induced the injury of H9C2 cardiomyocytes by H/R method, resulting in the accumulation of LDH and MDA and the decrease of SOD activity. Curcumin intervention significantly downregulated the transcription of NICD and the mRNA levels of its downstream signals (Hes-1, Hes-5, Hey-1, etc.), reversing the increase of ROS levels and decrease of antioxidant enzyme activity induced by H/R. In addition, Notch signaling activator (Jagged1) has a destructive effect on curcumin in alleviating H/R-induced cardiomyocyte injury, confirming that curcumin can play a myocardial protective role by inhibiting Notch signaling pathway.

## 10 Other signal pathways

Janus kinase/signal transduction and transcriptional activation factor (JAK/STAT) signals regulate communication between transmembrane receptors and the nucleus. There are more than 50 kinds of cytokines involved in the signal transduction, which play an important role in cell homeostasis, survival metabolism and immune response. Binding of the receptor to the extracellular ligand activates tyrosine phosphorylation of the JAK receptor and recruits STAT proteins, promoting phosphorylated STATs dimerization and translocation into the nucleus to regulate transcription of related target genes (Xue et al., 2023). Abdelsamia et al. (2019) injected STZ intraperitoneally into rats to establish a diabetic myocardial injury model. Curcumin treatment significantly reduced JAK2 and STAT3 phosphorylation levels in diabetic rats, alleviated the increased levels of NF-κB and IL-6, respectively reduced cTnI, CK-MB and TGF-β1 levels by 68.88%, 32.06% and 35.33%, and significantly improved STZ-induced cardiac injury in type 1 diabetic rats. Liao et al. (2021) established an I/R rat model by means of coronary artery ligation. Studies have shown that curcumin hydrogel inhibits ROS levels by enhancing the activities of SOD, CAT, GPX and GR, and significantly restores the activities of Ca^2+^ -ATPase and Na^+^-K^+^ -ATPase in rat myocardium. The JAK2/STAT3 inhibitor AG490 completely reversed the above changes, confirming the key role of JAK/STAT signaling pathway in the myocardial protection function of curcumin.

Silent information adjustment factor1 (SIRT)/FOXO signaling pathways are involved in the regulation of cell metabolism, apoptosis, autophagy, and oxidative stress, and other important biological processes. They play a protective function of cardiovascular, nervous and endocrine systems (Orea-Soufi et al., 2022; Singh and Ubaid, 2020; Ding et al., 2024). In the oxidative stress model of porcine renal epithelial cells (PK-15) constructed using zealenone (ZEA) induction (Cui et al., 2023), ZEA inhibited PK-15 cell viability and induced ROS overproduction in a concentration-dependent manner. Curcumin treatment significantly reversed the above changes, strongly inhibited the decrease of SIRT1 mRNA expression and the increase of FOXO1 mRNA expression, significantly increased the activity of SOD and CAT in cells, and effectively alleviated ZEA-induced oxidative stress. Ren et al. (2020) used STZ combined with high-sugar and high-fat diet to construct a rat diabetes model. The results showed that curcumin intervention significantly improved the collagen deposition around the cardiovascular system, enhanced the pumping ability of the heart, significantly upregulated the expression of SOD, NQO1 and Nrf2, inhibited the decrease of the ratio of Bcl-2/Bax, reversed the decrease of the phosphorylation levels of Sirt1, PI3K and Akt and the increase of the acetylation level of Foxo1 in cardiomyocytes. Moreover, the specific inhibitors EX527(Sirt1 inhibitor) and LY294002(PI3K inhibitor) partially reversed the above changes, further confirming the key role of SIRT/FOXO and PI3K/Akt signaling pathways in curcumin’s regulation of oxidative stress and apoptosis in diabetic cardiomyopathy.

## 11 Conclusion and perspectives

Oxidative stress is easy to cause damage to biological macromolecules, which is a key factor in inducing many malignant diseases such as atherosclerosis, chronic obstructive pulmonary disease, Alzheimer’s disease and cancer, and seriously affects the quality of life and survival rate of human beings (Forman and Zhang, 2021). At present, a series of natural plant active ingredients have been studied and developed into mature antioxidant drugs. Curcumin has demonstrated significant research and development potential, and its anti-oxidative stress mechanisms, both *in vivo* and *in vitro*, are presented in Tables 1, 2. This paper systematically describes its antioxidant mechanism from the perspective of signaling pathways, mainly including Keap1-Nrf2/ARE, NF-κB, NOX and other pathways. However, the specific regulatory mechanisms of curcumin for some pathways and the complex crosstalk mechanism between signaling pathways are not fully understood, and most of them remain in the detection stage of gene or protein expression. It is still necessary to use gene knockout, molecular docking and other technical means, and use genomics, transcriptomics, proteomics, metabolomics or multi-omics analysis to further study the anti-oxidative stress target and the complex potential relationship between signaling pathways or between pathways and diseases. Curcumin acts synergistically with a variety of natural antioxidants. For instance, when combined with vitamin C, curcumin enhances the activity of endogenous antioxidant enzymes, which collectively scavenge free radicals and reduce immunosuppressant-induced hepatotoxicity (Hasan Khudhair et al., 2022). Curcumin, polydatin, and quercetin can counteract pro-inflammatory and pro-oxidative signals induced by both hyperglycemic and senescence conditions (Matacchione et al., 2022). Curcumin and resveratrol exhibit strong synergistic antioxidant effects, which offering potential for developing new combination therapies for endothelial dysfunction and other oxidative stress-related conditions (Zhou et al., 2021). Compared to conventional therapies, the combined use of curcumin with other natural antioxidants offers multiple advantages, including enhanced therapeutic efficacy, reduced side effects, overcoming drug resistance, and improved metabolic and immune regulation. These benefits address the limitations of single-target drugs and demonstrate broad potential for clinical applications. However, curcumin has poor water solubility, poor stability and low bioavailability, which seriously limits its potential for drug development and clinical application. At present, various methods have been adopted to solve these problems, such as constructing a curcumin nano drug delivery system (Chang et al., 2023), curcumin solid dispersion (Song W. et al., 2022) and curcumin metal complex (Prasad et al., 2021), or using curcumin as a lead compound to study its derivatives and analogues (Moetlediwa et al., 2023; Noureddin et al., 2019), thereby enhancing its performance and ensuring stable metabolism, which provides scientific guarantee for the research and development of curcumin and the expansion of its clinical application.

**TABLE 1:** curcumin anti-oxidative stress mechanism *in vivo* studies.

Signal pathways	Diseases model	Mechanism *in vivo* studies	References
Keap1-Nrf2/ARE	ICH	upregulated the expression of HO-1, NQO1, Gpx4 and other antioxidant proteins	Duan et al. (2022)
	cardiac fibrosis	significantly enhanced the activity of eNOS, upregulated the expression of endothelial cell markers	Chen et al. (2022)
	kidney injury	increased the expression levels of Nrf2 and its downstream genes CAT, SOD1, NQO1 and GCLC	Wang et al. (2022)
	lung injury	promoted the expression and nuclear translocation of Nrf2 protein, and increased the expression of its downstream related proteins such as NQO1, GCLC and HO-1	Xu et al. (2024)
	depression	reduced MDA, 4-hydroxynonenal (4-HNE) and serum corticosterone levels, and reduced DNA oxidative damage	Liao et al. (2020)
NF-κB	myocardial injury	decreased the expressions of HIF-1α, $O_{2}^{\cdot -}$ , NF-κB p65 and endoplasmic reticulum stress markers (Grp78 and CHOP) in myocardial tissue of mice exposed to IH	Moulin et al. (2020)
	myocardial injury	decreased the contents of cTnI and CK-MB in serum	Chen et al. (2023)
	acute gout	downregulated the mRNA expressions of inflammatory cytokines IL-1β, IL-6, TNF-a and COX-2	Chen et al. (2019)
	liver injury	enhanced SOD,CAT activities and GSH levels in rats, and the high levels of expression of 4-HNE, NF-κB, Bax and Apaf-1 under Cyp were reversed	Hussain et al. (2023)
	renal dysfunction	blocked the activation of NF-κB pathway, and decreased the expression levels of IL-1, IL-6 and TNF-a	Eldesoqui et al. (2023)
NOX	hypertensive	reversed the upregulation of gp91phox expression under metal exposure, intracellular GSH levels and their redox ratio increased	Tubsakul et al. (2021)
	Parkinson’s disease	reversed the upregulation of p47phox, p67phox and gp91phox, reduced the activity of NF-κB protein, and increased the level of GSH and its redox ratio	Sharma and Nehru, (2018)
MAPK	hypertensive	hexahydrocurcumin enhanced the activities of eNOS, p-eNOS and SOD, inhibited the expression of p-ERK1/2, p-JNK and p-p38 proteins	Panthiya et al. (2022b)
	heart injury	enhanced the activities of GSH, SOD and CAT, decreased the phosphorylation levels of p38MAPK, JNK and ERK1/2, upregulated anti-apoptotic factors Bcl-2 and BAG-1	Sarawi et al. (2021)
PI3K/Akt	MIRI	upregulated the expression of mTOR, PI3K and p-Akt, increased the expression of Bcl-2/Bax, decreased the level of autophagy, and significantly increased the activity of GSH and SOD	Wu et al. (2021)
	heart injury	enhanced SOD activity, reduced $O_{2}^{\cdot -}$ and MDA levels, and offset the DOX-induced decline in AKT and mTOR phosphorylation levels	Yu et al. (2020b)
	polycystic ovary syndrome	nanomorcumin restored PI3K/AKT/mTOR to normal levels, and increased GSH and SOD activities	Abuelezz et al. (2020)
AMPK	autoimmune encephalomyelitis	increased the levels of p-AMPK Thr172 and SIRT1	Sadek et al. (2024)
Wnt/β-Catenin	I/R	reversed the increase of β-Catenin and p-GSK3β, increased the activity of SOD and GSH-Px	Zhu et al. (2024)
	diabetic glomerular injury	restored the inhibitory effect of high glucose on the expression of Wnt5a and nuclear β-catenin protein, inhibited the increased expression of TGF-β1 and fibronectin	Ho et al. (2016)
JAK/STAT	diabetic myocardial injury	reduced JAK2 and STAT3 phosphorylation levels, alleviated the increased levels of NF-κB and IL-6, reduced cTnI, CK-MB and TGF-β1 levels	Abdelsamia et al. (2019)
	I/R	curcumin hydrogel enhanced the activities of SOD, CAT, GPX and GR, and restored the activities of Ca^2+^ -ATPase and Na^+^-K^+^ -ATPase in rat myocardium	Liao et al. (2021)
SIRT/FOXO	diabetes	reversed the decrease of the phosphorylation levels of Sirt1, PI3K and Akt and the increase of the acetylation level of Foxo1 in cardiomyocytes	Ren et al. (2020)

**TABLE 2:** curcumin anti-oxidative stress mechanism *in vitro* studies.

Signal pathways	Cell categories	Mechanism *in vitro* studies	Reference
Keap1-Nrf2/ARE	H9C2 cardiocytes	enhanced the activities of antioxidant enzymes HO-1, SOD and Gpx, and inhibited the production of ROS	Wu et al. (2022)
	human corneal endothelial cells	maintained the original form of the cells and promoted their proliferation and differentiation	Guo et al. (2021)
NF-κB	sepsis H9C2 cells	inhibited NF-κB phosphorylation by blocking dimerization, downregulated the expression of TNF-α and IL-6	Chen et al. (2023)
	THP-1 cells	inhibited the degradation of IκBα protein, and controlled the expression levels of p-p65, p-p50, p65, p50	Chen et al. (2019)
	bovine mammary epithelial cell	enhanced the activity of T-SOD and GSH, inhibited the levels of NF-κB subunits p65 and p50, and downregulated the expression of IL-8, IL-1β, IL-6 and TNF-a	Li et al. (2021)
NOX	aortic VSMCs	hexahydrocurcumin reversed the upregulation of NOX1 and NOX4 expression, enhanced the activity of PPAR-γ and its coactivator 1α(PGC-1α)	Panthiya et al. (2023a)
	VSMCs	controlled the overexpression of TLR4 and the activation of downstream NOX signaling pathway, inhibited the upregulated expression of p22phox mRNA and the increased secretion of TNF-α and IL-1	Zhai et al. (2015)
	human intestinal cell	reduced NOX activity, blocked nuclear translocation of NF-κB, and inhibited transcription and translation levels of multiple pro-inflammatory cytokines	Gupta et al. (2021)
	H9C2 embryonic rat cardiogenic cells	inhibited the increase of Rac1 activity and the expression of p22phox, p47phox, p67phox and gp91phox induced by PA exposure	Li et al. (2017)
MAPK	H9c2 cells	downregulated the expression levels of p-ERK1/2, p-JNK and endoplasmic reticulum stress markers (GRP78, CHOP)	Wei et al. (2019)
	SHSY5Y cells	reduced the phosphorylation levels of JNK and ERK proteins, and destroyed co-localization effect of P-JNK-SUMO-1	Buccarello et al. (2020)
PI3K/Akt	Cardiomyocytes	tetrahydrocurcumin reversed the H/R-induced increase in Bax/Bcl-2 expression, downregulated the expression of Beclin-1, and enhanced intracellular SOD and CAT activities	Chen et al. (2021)
	myofibroblasts	promoted the demethylation of PTEN and upregulated its expression, further inhibited the signaling pathway	Zhuang et al. (2019)
AMPK	H9C2 cells	nano-curcumin reversed the decreased level of p-AMPK and the increased levels of downstream targets p-mTORC1 and p-p70S6K in PA-induced state	Zhang et al. (2019)
	human umbilical vein endothelial cells	reversed the decline of p-AMPK and the increase of p-mTOR and p-p70S6K in ox-LDL -induced state, and upregulated the expression of LC3-II protein	Zhao et al. (2021)
	BNLCL.2 cell	restored the expression of PPAR-α to the physiological level, upregulated AMPK mRNA expression, downregulated mTOR mRNA expression, and enhanced GSH activity	Kong et al. (2020)
	RAW264.7 cells	negatively regulated the expression of osteoclast genes to protect bone loss	Tanaka et al. (2023)
Wnt/β-Catenin	Caco-2 cells	upregulated the expression of NKD2 through DNA demethylation to inhibit the signaling pathway	Zhu et al. (2024)
	non-small cell lung cancer cell line A549	inhibited the expression of β-Catenin, p-GSK3β and its downstream targets cyclinD1 and c-Myc	Wang et al. (2018)
	rat osteoblasts	reversed the down expression of Wnt5a and β-Catenin, and upregulated the expression of alkaline phosphatase	Zhao et al. (2022)
Notch	astrocytes	decreased the expression of Notch1, Hes1 and Bax proteins	Gao et al. (2019)
	H9C2 cardiomyocytes	downregulated the transcription of NICD and the mRNA levels of its downstream signals (Hes-1, Hes-5, Hey-1, etc.)	Zhu et al. (2019)
SIRT/FOXO	porcine renal epithelial cells	inhibited the decrease of SIRT1 mRNA expression and the increase of FOXO1 mRNA expression, and increased the activity of SOD and CAT in cells	Cui et al. (2023)

## References

[B1] Abdelsamia E. M. Khaleel S. A. Balah A. Abdel Baky N. A. (2019). Curcumin augments the cardioprotective effect of metformin in an experimental model of type I diabetes mellitus; Impact of Nrf2/HO-1 and JAK/STAT pathways. Biomed. Pharmacother. 109, 2136–2144. 10.1016/j.biopha.2018.11.064 30551471

[B2] Abuelezz N. Z. Shabana M. E. Abdel-Mageed H. M. Rashed L. Morcos G. N. B. (2020). Nanocurcumin alleviates insulin resistance and pancreatic deficits in polycystic ovary syndrome rats: insights on PI3K/AkT/mTOR and TNF-α modulations. Life Sci. 256, 118003. 10.1016/j.lfs.2020.118003 32589998

[B3] Barreca M. M. Alessandro R. Corrado C. (2023). Effects of flavonoids on cancer, cardiovascular and neurodegenerative diseases: role of NF-κB signaling pathway. Int. J. Mol. Sci. 24 (11), 9236. 10.3390/ijms24119236 37298188 PMC10252664

[B4] Buccarello L. Dragotto J. Iorio F. Hassanzadeh K. Corbo M. Feligioni M. (2020). The pivotal role of SUMO-1-JNK-Tau axis in an *in vitro* model of oxidative stress counteracted by the protective effect of curcumin. Biochem. Pharmacol. 178, 114066. 10.1016/j.bcp.2020.114066 32502496

[B5] Canovas B. Nebreda A. R. (2021). Diversity and versatility of p38 kinase signalling in health and disease. Nat. Rev. Mol. Cell Biol. 22 (5), 346–366. 10.1038/s41580-020-00322-w 33504982 PMC7838852

[B6] Chang R. Chen L. Qamar M. Wen Y. Li L. Zhang J. (2023). The bioavailability, metabolism and microbial modulation of curcumin-loaded nanodelivery systems. Adv. Colloid Interface Sci. 318, 102933. 10.1016/j.cis.2023.102933 37301064

[B7] Chen B. Li H. Ou G. Ren L. Yang X. Zeng M. (2019). Curcumin attenuates MSU crystal-induced inflammation by inhibiting the degradation of IκBα and blocking mitochondrial damage. Arthritis Res. Ther. 21 (1), 193. 10.1186/s13075-019-1974-z 31455356 PMC6712780

[B8] Chen D. Wang H. Cai X. (2023). Curcumin interferes with sepsis-induced cardiomyocyte apoptosis via TLR1 inhibition. Rev. Port. Cardiol. 42 (3), 209–221. 10.1016/j.repc.2023.01.013 36702348

[B9] Chen X. Chen C. X. Gao Z. Chen X. X. Hu J. Zhou H. (2022). Curcumin improves cardiac fibrosis by inhibiting endothelial-mesenchymal transition through NRF2-DDAH-ADMA-NO pathway. Zhongguo Zhong Yao Za Zhi 47 (3), 745–752. 10.19540/j.cnki.cjcmm.20211019.701 35178958

[B10] Chen X. Xie Q. Zhu Y. Xu J. Lin G. Liu S. (2021). Cardio-protective effect of tetrahydrocurcumin, the primary hydrogenated metabolite of curcumin *in vivo* and *in vitro*: induction of apoptosis and autophagy via PI3K/AKT/mTOR pathways. Eur. J. Pharmacol. 911, 174495. 10.1016/j.ejphar.2021.174495 34555398

[B11] Cicenas J. Zalyte E. Rimkus A. Dapkus D. Noreika R. Urbonavicius S. (2017). JNK, p38, ERK, and SGK1 inhibitors in cancer. Cancers (Basel) 10 (1), 1. 10.3390/cancers10010001 29267206 PMC5789351

[B12] Cui H. J. Lu C. T. Pan L. Q. Hu H. Zhong P. Y. Zhu J. Y. (2023). Curcumin alleviates zearalenone-induced oxidative injury of porcine renal epithelial cells through the SIRT1/FOXO1 pathway. Sci. Agric. Sin. 56 (05), 1007–1018. [In Chinese].

[B13] Daverey A. Agrawal S. K. (2020). Curcumin protects against white matter injury through NF-κB and Nrf2 cross talk. J. Neurotrauma 37 (10), 1255–1265. 10.1089/neu.2019.6749 31914858

[B14] Ding X. Zhu C. Wang W. Li M. Ma C. Gao B. (2024). SIRT1 is a regulator of autophagy: implications for the progression and treatment of myocardial ischemia-reperfusion. Pharmacol. Res. 199, 106957. 10.1016/j.phrs.2023.106957 37820856

[B15] Duan C. Wang H. Jiao D. Geng Y. Wu Q. Yan H. (2022). Curcumin restrains oxidative stress of after intracerebral hemorrhage in rat by activating the Nrf2/HO-1 pathway. Front. Pharmacol. 13, 889226. 10.3389/fphar.2022.889226 35571134 PMC9092178

[B16] Eldesoqui M. Ahmed M. E. Abdel-Kareem M. A. Badawy M. M. Dawood A. F. Mohamed A. S. (2023). Curcumin mitigates malathion-induced renal injury: suppression of apoptosis and modulation of NF-κβ/TNF-α and Nrf2, and HO-1 signaling. Metabolites 13 (11), 1117. 10.3390/metabo13111117 37999213 PMC10673029

[B17] Fallahnezhad S. Ghorbani-Taherdehi F. Sahebkar A. Nadim A. Kafashzadeh M. Kafashzadeh M. (2023). Potential neuroprotective effect of nanomicellar curcumin on learning and memory functions following subacute exposure to bisphenol A in adult male rats. Metab. Brain Dis. 38 (8), 2691–2720. 10.1007/s11011-023-01257-9 37843661

[B18] Farzaei M. H. Zobeiri M. Parvizi F. El-Senduny F. F. Marmouzi I. Coy-Barrera E. (2018). Curcumin in liver diseases: a systematic review of the cellular mechanisms of oxidative stress and clinical perspective. Nutrients 10 (7), 855. 10.3390/nu10070855 29966389 PMC6073929

[B19] Feng K. Ge Y. Chen Z. Li X. Liu Z. Li X. (2019). Curcumin inhibits the PERK-eIF2α-CHOP pathway through promoting SIRT1 expression in oxidative stress-induced rat chondrocytes and ameliorates osteoarthritis progression in a rat model. Oxid. Med. Cell Longev. 2019, 8574386. 10.1155/2019/8574386 31223428 PMC6541984

[B20] Forman H. J. Zhang H. (2021). Targeting oxidative stress in disease: promise and limitations of antioxidant therapy. Nat. Rev. Drug Discov. 20 (9), 689–709. 10.1038/s41573-021-00233-1 34194012 PMC8243062

[B21] Forrester S. J. Kikuchi D. S. Hernandes M. S. Xu Q. Griendling K. K. (2018). Reactive oxygen species in metabolic and inflammatory signaling. Circ. Res. 122 (6), 877–902. 10.1161/CIRCRESAHA.117.311401 29700084 PMC5926825

[B22] Gao F. Zhao L. Yang Y. L. (2019). Effect of curcumin on spinal cord astrocyte injury induced by H_2_O_2_ through Notch signaling pathway. Chin. J. Immunol. 35 (02), 165–170. [In Chinese].

[B23] Guo S. P. Chang H. C. Lu L. S. Liu D. Z. Wang T. J. (2021). Activation of kelch-like ECH-associated protein 1/nuclear factor erythroid 2-related factor 2/antioxidant response element pathway by curcumin enhances the anti-oxidative capacity of corneal endothelial cells. Biomed. Pharmacother. 141, 111834. 10.1016/j.biopha.2021.111834 34153850

[B24] Gupta K. B. Mantha A. K. Dhiman M. (2021). Mitigation of gliadin-induced inflammation and cellular damage by curcumin in human intestinal cell lines. Inflammation 44 (3), 873–889. 10.1007/s10753-020-01383-x 33394186

[B25] Hamdy S. Elshopakey G. E. Risha E. F. Rezk S. Ateya A. I. Abdelhamid F. M. (2024). Curcumin mitigates gentamicin induced-renal and cardiac toxicity via modulation of Keap1/Nrf2, NF-κB/iNOS and Bcl-2/BAX pathways. Food Chem. Toxicol. 183, 114323. 10.1016/j.fct.2023.114323 38056816

[B26] Hasan Khudhair D. Al-Gareeb A. I. Al-Kuraishy H. M. El-Kadem A. H. Elekhnawy E. Negm W. A. (2022). Combination of vitamin C and curcumin safeguards against methotrexate-induced acute liver injury in mice by synergistic antioxidant effects. Front. Med. 9, 866343. 10.3389/fmed.2022.866343 PMC9047671 35492324

[B27] Hatamipour M. Johnston T. P. Sahebkar A. (2018). One molecule, many targets and numerous effects: the pleiotropy of curcumin lies in its chemical structure. Curr. Pharm. Des. 24 (19), 2129–2136. 10.2174/1381612824666180522111036 29788873

[B28] Hawkins C. L. Davies M. J. (2019). Detection, identification, and quantification of oxidative protein modifications. J. Biol. Chem. 294 (51), 19683–19708. 10.1074/jbc.REV119.006217 31672919 PMC6926449

[B29] He M. Li Y. Zhang L. Li L. Shen Y. Lin L. (2014). Curcumin suppresses cell proliferation through inhibition of the Wnt/β-catenin signaling pathway in medulloblastoma. Oncol. Rep. 32 (1), 173–180. 10.3892/or.2014.3206 24858998

[B30] He Y. Sun M. M. Zhang G. G. Yang J. Chen K. S. Xu W. W. (2021). Targeting PI3K/Akt signal transduction for cancer therapy. Signal Transduct. Target Ther. 6 (1), 425. 10.1038/s41392-021-00828-5 34916492 PMC8677728

[B31] Herzig S. Shaw R. J. (2018). AMPK: guardian of metabolism and mitochondrial homeostasis. Nat. Rev. Mol. Cell Biol. 19 (2), 121–135. 10.1038/nrm.2017.95 28974774 PMC5780224

[B32] Ho C. Hsu Y. C. Lei C. C. Mau S. C. Shih Y. H. Lin C. L. (2016). Curcumin rescues diabetic renal fibrosis by targeting superoxide-mediated Wnt signaling pathways. Am. J. Med. Sci. 351 (3), 286–295. 10.1016/j.amjms.2015.12.017 26992258

[B33] Hussain S. Ashafaq M. Alshahrani S. Bokar I. A. M. Siddiqui R. Alam M. I. (2023). Hepatoprotective effect of curcumin nano-lipid carrier against cypermethrin toxicity by countering the oxidative, inflammatory, and apoptotic changes in wistar rats. Molecules 28 (2), 881. 10.3390/molecules28020881 36677938 PMC9864069

[B34] Iside C. Scafuro M. Nebbioso A. Altucci L. (2020). SIRT1 activation by natural phytochemicals: an overview. Front. Pharmacol. 11, 1225. 10.3389/fphar.2020.01225 32848804 PMC7426493

[B35] Ji L. L. Yeo D. (2021). Oxidative stress: an evolving definition. Fac. Rev. 10, 13. 10.12703/r/10-13 33659931 PMC7894272

[B36] Kciuk M. Gielecińska A. Budzinska A. Mojzych M. Kontek R. (2022). Metastasis and MAPK pathways. Int. J. Mol. Sci. 23 (7), 3847. 10.3390/ijms23073847 35409206 PMC8998814

[B37] Kishi S. Nagasu H. Kidokoro K. Kashihara N. (2024). Oxidative stress and the role of redox signalling in chronic kidney disease. Nat. Rev. Nephrol. 20 (2), 101–119. 10.1038/s41581-023-00775-0 37857763

[B38] Kong D. Zhang Z. Chen L. Huang W. Zhang F. Wang L. (2020). Curcumin blunts epithelial-mesenchymal transition of hepatocytes to alleviate hepatic fibrosis through regulating oxidative stress and autophagy. Redox Biol. 36, 101600. 10.1016/j.redox.2020.101600 32526690 PMC7287144

[B39] Kopan R. Ilagan M. X. (2009). The canonical Notch signaling pathway: unfolding the activation mechanism. Cell 137 (2), 216–233. 10.1016/j.cell.2009.03.045 19379690 PMC2827930

[B40] Kovall R. A. Gebelein B. Sprinzak D. Kopan R. (2017). The canonical Notch signaling pathway: structural and biochemical insights into shape, sugar, and force. Dev. Cell 41 (3), 228–241. 10.1016/j.devcel.2017.04.001 28486129 PMC5492985

[B41] Leyane T. S. Jere S. W. Houreld N. N. (2021). Cellular signalling and photobiomodulation in chronic wound repair. Int. J. Mol. Sci. 22 (20), 11223. 10.3390/ijms222011223 34681882 PMC8537491

[B42] Li J. Zhou Y. Zhang W. Bao C. Xie Z. (2017). Relief of oxidative stress and cardiomyocyte apoptosis by using curcumin nanoparticles. Colloids Surf. B Biointerfaces 153, 174–182. 10.1016/j.colsurfb.2017.02.023 28237821

[B43] Li R. Fang H. Shen J. Jin Y. Zhao Y. Wang R. (2021). Curcumin alleviates LPS-induced oxidative stress, inflammation and apoptosis in bovine mammary epithelial cells via the NFE2L2 signaling pathway. Toxins (Basel). 13 (3), 208. 10.3390/toxins13030208 33809242 PMC7999830

[B44] Li X. Chen Y. Mao Y. Dai P. Sun X. Zhang X. (2020b). Curcumin protects osteoblasts from oxidative stress-induced dysfunction via GSK3β-Nrf2 signaling pathway. Front. Bioeng. Biotechnol. 8, 625. 10.3389/fbioe.2020.00625 32612986 PMC7308455

[B45] Li X. Lin H. Zhang X. Jaspers R. T. Yu Q. Ji Y. (2021a). Notoginsenoside R1 attenuates oxidative stress-induced osteoblast dysfunction through JNK signalling pathway. J. Cell Mol. Med. 25 (24), 11278–11289. 10.1111/jcmm.17054 34786818 PMC8650043

[B46] Li X. Sung P. Zhang D. Yan L. (2023). Curcumin *in vitro* neuroprotective Effects Are Mediated by p62/keap-1/Nrf2 and PI3K/AKT Signaling Pathway and Autophagy Inhibition. Physiol. Res. 72 (4), 497–510. 10.33549/physiolres.935054 37795892 PMC10634561

[B47] Liao C. L. Liu Y. Huang M. Z. Liu H. Y. Ye Z. L. Su Q. (2021). Myocardial ischemia reperfusion injury is alleviated by curcumin-peptide hydrogel via upregulating autophagy and protecting mitochondrial function. Stem Cell Res. Ther. 12 (1), 89. 10.1186/s13287-020-02101-y 33509263 PMC7842017

[B48] Liao D. Lv C. Cao L. Yao D. Wu Y. Long M. (2020). Curcumin attenuates chronic unpredictable mild stress-induced depressive-like behaviors via restoring changes in oxidative stress and the activation of Nrf2 signaling pathway in rats. Oxid. Med. Cell Longev. 2020, 9268083. 10.1155/2020/9268083 33014280 PMC7520007

[B49] Liu Z. Cui C. Xu P. Dang R. Cai H. Liao D. (2017). Curcumin activates AMPK pathway and regulates lipid metabolism in rats following prolonged clozapine exposure. Front. Neurosci. 11, 558. 10.3389/fnins.2017.00558 29046626 PMC5632657

[B50] Luo G. Wang R. Zhou H. Liu X. (2021). ALDOA protects cardiomyocytes against H/R-induced apoptosis and oxidative stress by regulating the VEGF/Notch 1/Jagged 1 pathway. Mol. Cell Biochem. 476 (2), 775–783. 10.1007/s11010-020-03943-z 33089381

[B51] Matacchione G. Valli D. Silvestrini A. Giuliani A. Sabbatinelli J. Giordani C. (2022). Curcumin, polydatin and quercetin synergistic activity protects from high-glucose-induced inflammation and oxidative stress. Antioxidants 11 (6), 1037. 10.3390/antiox11061037 35739934 PMC9220232

[B52] Meurette O. Mehlen P. (2018). Notch signaling in the tumor microenvironment. Cancer Cell 34 (4), 536–548. 10.1016/j.ccell.2018.07.009 30146333

[B53] Moetlediwa M. T. Ramashia R. Pheiffer C. Titinchi S. J. J. Mazibuko-Mbeje S. E. Jack B. U. (2023). Therapeutic effects of curcumin derivatives against obesity and associated metabolic complications: a review of *in vitro* and *in vivo* studies. Int. J. Mol. Sci. 24 (18), 14366. 10.3390/ijms241814366 37762669 PMC10531575

[B54] Mossmann D. Park S. Hall M. N. (2018). mTOR signalling and cellular metabolism are mutual determinants in cancer. Nat. Rev. Cancer 18 (12), 744–757. 10.1038/s41568-018-0074-8 30425336

[B55] Moulin S. Arnaud C. Bouyon S. Pépin J. L. Godin-Ribuot D. Belaidi E. (2020). Curcumin prevents chronic intermittent hypoxia-induced myocardial injury. Ther. Adv. Chronic Dis. 11, 2040622320922104. 10.1177/2040622320922104 32637058 PMC7315663

[B56] Mukherjee T. Kumar N. Chawla M. Philpott D. J. Basak S. (2024). The NF-κB signaling system in the immunopathogenesis of inflammatory bowel disease. Sci. Signal 17 (818), eadh1641. 10.1126/scisignal.adh1641 38194476

[B57] Nile A. H. Hannoush R. N. (2016). Fatty acylation of Wnt proteins. Nat. Chem. Biol. 12 (2), 60–69. 10.1038/nchembio.2005 26784846

[B58] Niture S. K. Kaspar J. W. Shen J. Jaiswal A. K. (2010). Nrf2 signaling and cell survival. Toxicol. Appl. Pharmacol. 244 (1), 37–42. 10.1016/j.taap.2009.06.009 19538984 PMC2837794

[B59] Noureddin S. A. El-Shishtawy R. M. Al-Footy K. O. (2019). Curcumin analogues and their hybrid molecules as multifunctional drugs. Eur. J. Med. Chem. 182, 111631. 10.1016/j.ejmech.2019.111631 31479974

[B60] Nusse R. Clevers H. (2017). Wnt/β-Catenin signaling, disease, and emerging therapeutic modalities. Cell 169 (6), 985–999. 10.1016/j.cell.2017.05.016 28575679

[B61] Ogboo B. C. Grabovyy U. V. Maini A. Scouten S. van der Vliet A. Mattevi A. (2022). Architecture of the NADPH oxidase family of enzymes. Redox Biol. 52, 102298. 10.1016/j.redox.2022.102298 35334249 PMC8956913

[B62] Orea-Soufi A. Paik J. Bragança J. Donlon T. A. Willcox B. J. Link W. (2022). FOXO transcription factors as therapeutic targets in human diseases. Trends Pharmacol. Sci. 43 (12), 1070–1084. 10.1016/j.tips.2022.09.010 36280450 PMC12194985

[B63] Panthiya L. Tocharus J. Chaichompoo W. Suksamrarn A. Tocharus C. (2023a). Hexahydrocurcumin mitigates angiotensin II-induced proliferation, migration, and inflammation in vascular smooth muscle cells. EXCLI J. 22, 466–481. 10.17179/excli2023-6124 37534221 PMC10391613

[B64] Panthiya L. Tocharus J. Onsa-Ard A. Chaichompoo W. Suksamrarn A. Tocharus C. (2022b). Hexahydrocurcumin ameliorates hypertensive and vascular remodeling in L-NAME-induced rats. Biochim. Biophys. Acta Mol. Basis Dis. 1868 (3), 166317. 10.1016/j.bbadis.2021.166317 34883248

[B65] Patel S. S. Acharya A. Ray R. S. Agrawal R. Raghuwanshi R. Jain P. (2020). Cellular and molecular mechanisms of curcumin in prevention and treatment of disease. Crit. Rev. Food Sci. Nutr. 60 (6), 887–939. 10.1080/10408398.2018.1552244 30632782

[B66] Peng M. L. Fu Y. Wu C. W. Zhang Y. Ren H. Zhou S. S. (2022). Signaling pathways related to oxidative stress in diabetic cardiomyopathy. Front. Endocrinol. (Lausanne) 13, 907757. 10.3389/fendo.2022.907757 35784531 PMC9240190

[B67] Prasad S. DuBourdieu D. Srivastava A. Kumar P. Lall R. (2021). Metal-curcumin complexes in therapeutics: an approach to enhance pharmacological effects of curcumin. Int. J. Mol. Sci. 22 (13), 7094. 10.3390/ijms22137094 34209461 PMC8268053

[B68] Pulido-Moran M. Moreno-Fernandez J. Ramirez-Tortosa C. Ramirez-Tortosa M. (2016). Curcumin and health. Molecules 21 (3), 264. 10.3390/molecules21030264 26927041 PMC6273481

[B69] Ren B. C. Zhang Y. F. Liu S. S. Cheng X. J. Yang X. Cui X. G. (2020). Curcumin alleviates oxidative stress and inhibits apoptosis in diabetic cardiomyopathy via Sirt1-Foxo1 and PI3K-Akt signalling pathways. J. Cell Mol. Med. 24 (21), 12355–12367. 10.1111/jcmm.15725 32961025 PMC7687015

[B70] Rhee S. G. (2006). Cell signaling. H2O2, a necessary evil for cell signaling. Science 312 (5782), 1882–1883. 10.1126/science.1130481 16809515

[B71] Rim E. Y. Clevers H. Nusse R. (2022). The Wnt pathway: from signaling mechanisms to synthetic modulators. Annu. Rev. Biochem. 91, 571–598. 10.1146/annurev-biochem-040320-103615 35303793

[B72] Russell J. O. Monga S. P. (2018). Wnt/β-Catenin signaling in liver development, homeostasis, and pathobiology. Annu. Rev. Pathol. 13, 351–378. 10.1146/annurev-pathol-020117-044010 29125798 PMC5927358

[B73] Sadek M. A. Rabie M. A. El Sayed N. S. Sayed H. M. Kandil E. A. (2024). Neuroprotective effect of curcumin against experimental autoimmune encephalomyelitis-induced cognitive and physical impairments in mice: an insight into the role of the AMPK/SIRT1 pathway. Inflammopharmacology 32 (2), 1499–1518. 10.1007/s10787-023-01399-3 38112964 PMC11006778

[B74] Sarawi W. S. Alhusaini A. M. Fadda L. M. Alomar H. A. Albaker A. B. Aljrboa A. S. (2021). Nano-curcumin prevents cardiac injury, oxidative stress and inflammation, and modulates TLR4/NF-κB and MAPK signaling in copper sulfate-intoxicated rats. Antioxidants (Basel) 10 (9), 1414. 10.3390/antiox10091414 34573046 PMC8469340

[B75] Schieber M. Chandel N. S. (2014). ROS function in redox signaling and oxidative stress. Curr. Biol. 24 (10), R453–R462. 10.1016/j.cub.2014.03.034 24845678 PMC4055301

[B76] Sharma N. Nehru B. (2018). Curcumin affords neuroprotection and inhibits α-synuclein aggregation in lipopolysaccharide-induced Parkinson's disease model. Inflammopharmacology 26 (2), 349–360. 10.1007/s10787-017-0402-8 29027056

[B77] Shin J. W. Chun K. S. Kim D. H. Kim S. J. Kim S. H. Cho N. C. (2020). Curcumin induces stabilization of Nrf2 protein through Keap1 cysteine modification. Biochem. Pharmacol. 173, 113820. 10.1016/j.bcp.2020.113820 31972171

[B78] Sies H. Berndt C. Jones D. P. (2017). Oxidative stress. Annu. Rev. Biochem. 86, 715–748. 10.1146/annurev-biochem-061516-045037 28441057

[B79] Silva-Gaona O. G. Hernández-Ortiz M. Vargas-Ortiz K. Ramírez-Emiliano J. Garay-Sevilla M. E. Encarnación-Guevara S. (2022). Curcumin prevents proteins expression changes of oxidative phosphorylation, cellular stress response, and lipid metabolism proteins in liver of mice fed a high-fructose diet. J. Proteomics 263, 104595. 10.1016/j.jprot.2022.104595 35490921

[B80] Singh V. Ubaid S. (2020). Role of silent information regulator 1 (SIRT1) in regulating oxidative stress and inflammation. Inflammation 43 (5), 1589–1598. 10.1007/s10753-020-01242-9 32410071

[B81] Song C. Liu B. Li H. Tang Y. Ge X. Liu B. (2022a). Protective effects of emodin on oxidized fish oil-induced metabolic disorder and oxidative stress through notch-Nrf2 crosstalk in the liver of Teleost *Megalobrama amblycephala* . Antioxidants (Basel) 11 (6), 1179. 10.3390/antiox11061179 35740076 PMC9219933

[B82] Song N. Thaiss F. Guo L. (2019). NFκB and kidney injury. Front. Immunol. 10, 815. 10.3389/fimmu.2019.00815 31040851 PMC6477040

[B83] Song W. Chen X. Dai C. Lin D. Pang X. Zhang D. (2022b). Comparative study of preparation, evaluation, and pharmacokinetics in beagle dogs of curcumin β-cyclodextrin inclusion complex, curcumin solid dispersion, and curcumin phospholipid complex. Molecules 27 (9), 2998. 10.3390/molecules27092998 35566349 PMC9102399

[B84] Tanaka M. Inoue H. Takahashi N. Uehara M. (2023). AMPK negatively regulates RANKL-induced osteoclast differentiation by controlling oxidative stress. Free Radic. Biol. Med. 205, 107–115. 10.1016/j.freeradbiomed.2023.05.033 37270032

[B85] Tandon A. Singh S. J. Gupta M. Singh N. Shankar J. Arjaria N. (2020). Notch pathway up-regulation via curcumin mitigates bisphenol-A (BPA) induced alterations in hippocampal oligodendrogenesis. J. Hazard Mater 392, 122052. 10.1016/j.jhazmat.2020.122052 32151947

[B86] Taniguchi K. Karin M. (2018). NF-κB, inflammation, immunity and cancer: coming of age. Nat. Rev. Immunol. 18 (5), 309–324. 10.1038/nri.2017.142 29379212

[B87] Trefts E. Shaw R. J. (2021). AMPK: restoring metabolic homeostasis over space and time. Mol. Cell 81 (18), 3677–3690. 10.1016/j.molcel.2021.08.015 34547233 PMC8549486

[B88] Tubita A. Lombardi Z. Tusa I. Dello Sbarba P. Rovida E. (2020). Beyond kinase activity: ERK5 nucleo-cytoplasmic shuttling as a novel target for anticancer therapy. Int. J. Mol. Sci. 21 (3), 938. 10.3390/ijms21030938 32023850 PMC7038028

[B89] Tubsakul A. Sangartit W. Pakdeechote P. Kukongviriyapan V. Apaijit K. Kukongviriyapan U. (2021). Curcumin mitigates hypertension, endothelial dysfunction and oxidative stress in rats with chronic exposure to lead and cadmium. Tohoku J. Exp. Med. 253 (1), 69–76. 10.1620/tjem.253.69 33473064

[B90] Vallée A. Lecarpentier Y. (2018a). Crosstalk between peroxisome proliferator-activated receptor gamma and the canonical WNT/β-Catenin pathway in chronic inflammation and oxidative stress during carcinogenesis. Front. Immunol. 9, 745. 10.3389/fimmu.2018.00745 29706964 PMC5908886

[B91] Vallée A. Lecarpentier Y. Vallée J. N. (2019b). Curcumin: a therapeutic strategy in cancers by inhibiting the canonical WNT/β-catenin pathway. J. Exp. Clin. Cancer Res. 38 (1), 323. 10.1186/s13046-019-1320-y 31331376 PMC6647277

[B92] Vermot A. Petit-Härtlein I. Smith S. M. E. Fieschi F. (2021). NADPH oxidases (NOX): an overview from discovery, molecular mechanisms to physiology and pathology. Antioxidants (Basel) 10 (6), 890. 10.3390/antiox10060890 34205998 PMC8228183

[B93] Wang J. Y. Wang X. Wang X. J. Zheng B. Z. Wang Y. Wang X. (2018). Curcumin inhibits the growth via Wnt/β-catenin pathway in non-small-cell lung cancer cells. Eur. Rev. Med. Pharmacol. Sci. 22 (21), 7492–7499. 10.26355/eurrev_201811_16290 30468498

[B94] Wang Y. Liu F. Zhou X. Liu M. Zang H. Liu X. (2022). Alleviation of oral exposure to aflatoxin B1-induced renal dysfunction, oxidative stress, and cell apoptosis in mice kidney by curcumin. Antioxidants (Basel) 11 (6), 1082. 10.3390/antiox11061082 35739979 PMC9219944

[B95] Wei W. Peng J. Li J. (2019). Curcumin attenuates hypoxia/reoxygenation-induced myocardial injury. Mol. Med. Rep. 20 (6), 4821–4830. 10.3892/mmr.2019.10742 31638219 PMC6854596

[B96] Winterbourn C. C. Kettle A. J. Hampton M. B. (2016). Reactive oxygen species and neutrophil function. Annu. Rev. Biochem. 85, 765–792. 10.1146/annurev-biochem-060815-014442 27050287

[B97] Wong S. C. Kamarudin M. N. A. Naidu R. (2021). Anticancer mechanism of curcumin on human glioblastoma. Nutrients 13 (3), 950. 10.3390/nu13030950 33809462 PMC7998496

[B98] Wu H. J. Zhang K. Ma J. J. Wang L. Zhuang Y. (2021). Mechanism of curcumin against myocardial ischaemia-reperfusion injury based on the P13K/Akt/mTOR signalling pathway. Eur. Rev. Med. Pharmacol. Sci. 25 (17), 5490–5499. 10.26355/eurrev_202109_26658 34533799

[B99] Wu J. S. Tsai H. D. Cheung W. M. Hsu C. Y. Lin T. N. (2016). PPAR-Γ ameliorates neuronal apoptosis and ischemic brain injury via suppressing NF-κB-Driven p22phox transcription. Mol. Neurobiol. 53 (6), 3626–3645. 10.1007/s12035-015-9294-z 26108185

[B100] Wu X. Zhou X. Lai S. Liu J. Qi J. (2022). Curcumin activates Nrf2/HO-1 signaling to relieve diabetic cardiomyopathy injury by reducing ROS *in vitro* and *in vivo* . FASEB J. 36 (9), e22505. 10.1096/fj.202200543RRR 35971779

[B101] Xia N. Tenzer S. Lunov O. Karl M. Simmet T. Daiber A. (2021). Regulation of NADPH oxidase-mediated superoxide production by acetylation and deacetylation. Front. Physiol. 12, 693702. 10.3389/fphys.2021.693702 34456745 PMC8387964

[B102] Xing L. Fang J. Zhu B. Wang L. Chen J. Wang Y. (2021). Astragaloside IV protects against podocyte apoptosis by inhibiting oxidative stress via activating PPARγ-Klotho-FoxO1 axis in diabetic nephropathy. Life Sci. 269, 119068. 10.1016/j.lfs.2021.119068 33476631

[B103] Xu G. Chen H. Cong Z. Wang R. Li X. Xie Y. (2024). Promotion of transcription factor EB-dependent autophagic process by curcumin alleviates arsenic-caused lung oxidative stress and inflammation in mice. J. Nutr. Biochem. 125, 109550. 10.1016/j.jnutbio.2023.109550 38141737

[B104] Xuan W. Khan M. Ashraf M. (2020). Extracellular vesicles from Notch activated cardiac mesenchymal stem cells promote myocyte proliferation and neovasculogenesis. Front. Cell Dev. Biol. 8, 11. 10.3389/fcell.2020.00011 32154243 PMC7047205

[B105] Xue C. Yao Q. Gu X. Shi Q. Yuan X. Chu Q. (2023). Evolving cognition of the JAK-STAT signaling pathway: autoimmune disorders and cancer. Signal Transduct. Target Ther. 8 (1), 204. 10.1038/s41392-023-01468-7 37208335 PMC10196327

[B106] Yang B. Yin C. Zhou Y. Wang Q. Jiang Y. Bai Y. (2019). Curcumin protects against methylmercury-induced cytotoxicity in primary rat astrocytes by activating the Nrf2/ARE pathway independently of PKCδ. Toxicology 425, 152248. 10.1016/j.tox.2019.152248 31330227 PMC6710134

[B107] Yu H. Lin L. Zhang Z. Zhang H. Hu H. (2020a). Targeting NF-κB pathway for the therapy of diseases: mechanism and clinical study. Signal Transduct. Target Ther. 5 (1), 209. 10.1038/s41392-020-00312-6 32958760 PMC7506548

[B108] Yu W. Qin X. Zhang Y. Qiu P. Wang L. Zha W. (2020b). Curcumin suppresses doxorubicin-induced cardiomyocyte pyroptosis via a PI3K/Akt/mTOR-dependent manner. Cardiovasc Diagn Ther. 10 (4), 752–769. 10.21037/cdt-19-707 32968631 PMC7487371

[B109] Zanotti S. Canalis E. (2016). Notch signaling and the skeleton. Endocr. Rev. 37 (3), 223–253. 10.1210/er.2016-1002 27074349 PMC4890266

[B110] Zhai H. J. Meng Z. Tao H. L. Bai Z. L. Yan C. Li L. (2015). Effects of curcumin on Toll-like receptor 4/NADPH oxidase/ROS signaling pathways and inflammatory cytokines release in endotoxin-induced vascular smooth muscle cells. J. Xi 'an Jiaot. Univ. Med. Ed. 36 (04), 543–548. [In Chinese].

[B111] Zhang H. Dong Q. Q. Shu H. P. Tu Y. C. Liao Q. Q. Yao L. J. (2023). Curcumin ameliorates focal segmental glomerulosclerosis by inhibiting apoptosis and oxidative stress in podocytes. Arch. Biochem. Biophys. 746, 109728. 10.1016/j.abb.2023.109728 37633586

[B112] Zhang J. Wang Y. Bao C. Liu T. Li S. Huang J. (2019). Curcumin-loaded PEG-PDLLA nanoparticles for attenuating palmitate-induced oxidative stress and cardiomyocyte apoptosis through AMPK pathway. Int. J. Mol. Med. 44 (2), 672–682. 10.3892/ijmm.2019.4228 31173176 PMC6605976

[B113] Zhang Z. B. Luo D. D. Xie J. H. Xian Y. F. Lai Z. Q. Liu Y. H. (2018). Curcumin's metabolites, tetrahydrocurcumin and octahydrocurcumin, possess superior anti-inflammatory effects *in vivo* through suppression of TAK1-NF-κb pathway. Front. Pharmacol. 9, 1181. 10.3389/fphar.2018.01181 30386242 PMC6199526

[B114] Zhao L. Luo R. Yu H. Li S. Yu Q. Wang W. (2021). Curcumin protects human umbilical vein endothelial cells against high oxidized low density lipoprotein-induced lipotoxicity and modulates autophagy. Iran. J. Basic Med. Sci. 24 (12), 1734–1742. 10.22038/IJBMS.2021.59969.13297 35432800 PMC8976913

[B115] Zhao R. S. Liang W. J. Deng W. C. Lin Z. J. Zeng X. M. Hong Y. C. (2022). Mechanism of curcumin in protecting osteoblast function through anti-oxidative stress. Nat. Prod. Res. Dev. 34 (08), 1311–1318. [In Chinese]. 10.16333/j.1001-6880.2022.8.005

[B116] Zhou P. Deng F. Yang Z. Cao C. Zhao H. Liu F. (2022). Ginsenoside Rb1 inhibits oxidative stress-induced ovarian granulosa cell injury through Akt-FoxO1 interaction. Sci. China Life Sci. 65 (11), 2301–2315. 10.1007/s11427-021-2080-x 35661967

[B117] Zhou X. Afzal S. Zheng Y. F. Münch G. Li C. G. (2021). Synergistic protective effect of curcumin and resveratrol against oxidative stress in endothelial EAhy926 cells. Evid. Based Complement. Altern. Med. 2021, 2661025. 10.1155/2021/2661025 PMC8434903 34518768

[B118] Zhu J. X. Dun Y. Wu W. Shen J. Zhang F. Zhang L. (2024). Curcumin suppresses the Wnt/β-catenin signaling pathway by inhibiting NKD2 methylation to ameliorate intestinal ischemia/reperfusion injury. Kaohsiung J. Med. Sci. 40 (2), 175–187. 10.1002/kjm2.12782 38010861 PMC11895583

[B119] Zhu P. Yang M. He H. Kuang Z. Liang M. Lin A. (2019). Curcumin attenuates hypoxia/reoxygenation-induced cardiomyocyte injury by downregulating Notch signaling. Mol. Med. Rep. 20 (2), 1541–1550. 10.3892/mmr.2019.10371 31257466 PMC6625400

[B120] Zhuang Z. Yu D. Chen Z. Liu D. Yuan G. Yirong N. (2019). Curcumin inhibits joint contracture through PTEN demethylation and targeting PI3K/Akt/mTOR pathway in myofibroblasts from human joint capsule. Evid. Based Complement. Altern. Med. 2019, 4301238. 10.1155/2019/4301238 PMC6712967 31511778

